# High Number of Previous *Plasmodium falciparum* Clinical Episodes Increases Risk of Future Episodes in a Sub-Group of Individuals

**DOI:** 10.1371/journal.pone.0055666

**Published:** 2013-02-06

**Authors:** Cheikh Loucoubar, Laura Grange, Richard Paul, Augustin Huret, Adama Tall, Olivier Telle, Christian Roussilhon, Joseph Faye, Fatoumata Diene-Sarr, Jean-François Trape, Odile Mercereau-Puijalon, Anavaj Sakuntabhai, Jean-François Bureau

**Affiliations:** 1 Institut Pasteur, Department Genome and Genetics, Unité de Génétique Fonctionnelle des Maladies Infectieuses, Paris, France; 2 Centre National de la Recherche Scientifique, Unité Recherche Associée 3012, Paris, France; 3 Institut Pasteur de Dakar, Unité d’Epidémiologie des Maladies Infectieuses, Dakar, Senegal; 4 Université Paris Descartes, Mathématiques Appliquées Paris 5- Unité Mixte de Recherche Centre National de la Recherche Scientifique 8145, Paris, France; 5 Ecole des Hautes Etudes en Santé Publique, Rennes, France; 6 Institute of Health & Science, Paris, France; 7 Institut de Recherche pour le Développement, Dakar, Unité de Pathogénie Afro-Tropicale (Unité Mixte de Recherche 198), Dakar, Senegal; 8 Institut Pasteur, Department of Parasitology, Unité d’Immunologie Moléculaire des Parasites, Paris, France; 9 Mahidol University, Systems Biology of Diseases Unit, Faculty of Science, Bangkok, Thailand; University of Copenhagen, Denmark

## Abstract

There exists great disparity in the number of clinical *P. falciparum* episodes among children of the same age and living in similar conditions. The epidemiological determinants of such disparity are unclear. We used a data-mining approach to explore a nineteen-year longitudinal malaria cohort study dataset from Senegal and identify variables associated with increased risk of malaria episodes. These were then verified using classical statistics and replicated in a second cohort. In addition to age, we identified a novel high-risk group of children in whom the history of *P. falciparum* clinical episodes greatly increased risk of further episodes. Age and a high number of previous falciparum clinical episodes not only play major roles in explaining the risk of *P. falciparum* episodes but also are risk factors for different groups of people. Combined, they explain the majority of falciparum clinical attacks. Contrary to what is widely believed, clinical immunity to *P. falciparum* does not *de facto* occur following many *P. falciparum* clinical episodes. There exist a sub-group of children who suffer repeated clinical episodes. In addition to posing an important challenge for population stratification during clinical trials, this sub-group disproportionally contributes to the disease burden and may necessitate specific prevention and control measures.

## Introduction

Malaria is a major public health problem responsible for the deaths of approximately 800,000 people every year (http://www.who.int/malaria/world_malaria_report_2010/en/index.html). It is estimated that up to 124 million people in Africa live in areas at risk of seasonal epidemic malaria, and many more in areas outside Africa, where *Plasmodium* spp. transmission is less intense. Immunity to *Plasmodium falciparum*, the aetiological agent of lethal tertian malaria, is non-sterilizing [1]. Current wisdom suggests that, under intense transmission, clinical immunity develops during childhood after many infections [1], [2], whereby the individual can tolerate non-negligible parasite densities without showing symptoms. Subsequently anti-parasite immunity, that enables control of parasite density, develops much more slowly [3], leading to a state of premunition, whereby individuals harbour chronic infections [4]. Continued exposure to the parasite is seemingly required to maintain such premunition [5]. Complete protection from further infections is rarely, if ever, achieved. Although the lack of sterilizing immunity undermines vaccine strategies for elimination of the parasite, inducing solid anti-parasite and clinical immunity would reduce mortality, morbidity and possibly transmission.

The Dielmo project is a longitudinal study, established in 1990 in Senegal, to better understand the acquisition of immunity to *P. falciparum* [6]. As classically recognized, the acquisition of immunity against *P. falciparum* clinical episodes increases with age and hence repeated exposure to the parasite [7], [8], [9]. However, this longitudinal survey also highlighted significant disparity of risk among children, with the number of clinical malaria episodes experienced during the first two years of life varying from 1 to 20 [8]. Further studies confirmed this result [10], [11]. Part of this variation is genetically determined [12], [13], but many contributing biological parameters remain obscure. The objective of this study is to search for other major risk factors that affect susceptibility to *P. falciparum* clinical episodes in this well-documented longitudinal cohort.

Risk factor analyses for identifying key determinants of the occurrence of clinical malaria have traditionally used classical statistical tools, such as logistic regression or Cox proportional hazard models, that detect variables affecting an entire population (global effects) after taking into account the impact of confounding variables. These parametric methods are, however, less efficient in detecting risk factors impacting only upon certain sub-groups of the population with particular characteristics (local effects), such as the age-dependent protection afforded by sickle cell trait against risk of clinical *P. falciparum* episodes [14]: the number of models to test increases exponentially with the number of variables with a risk of over-fitting for the more complex models. Different data mining approaches have been developed to overcome this problem of dimensionality.

We have recently evaluated the utility of the HyperCube® “reverse engineering” program for exploring large and complex epidemiological data sets in a hypothesis free manner [15]. This method implements an exhaustive, non-Euclidean, non-parametric approach, and thereby enables detection of different combinations of explanatory variables (referred to as “rules”) affecting occurrence of an outcome (dependent variable), in this case clinical malaria episodes. The aim of this approach is to characterize high risk groups within a study population based on local over-densities of clinical malaria episodes, and to identify risk factors. Here we apply this methodology and consider thirty-six explanatory variables to identify key factors underlying the risk of clinical *P. falciparum* episodes in Dielmo village. We replicate the analysis in a second family-based Senegalese cohort in Ndiop village and then validate the results using classical statistical tools.

## Results

### Characteristics of the Cohort

We studied the large dataset from the long-term epidemiological study of a family-based cohort followed in Dielmo village for 19 years (1990–2008). For each participant, the dependent variable was defined as a dichotomous trait “experiencing no *P. falciparum* clinical episodes (PFA) during the trimester” or “having at least one PFA during the trimester”. Thirty-six explanatory variables for association with the occurrence of PFA were considered (Tables 1 & 2). These variables defined the status of an individual, such as sex, age, genetic traits, his history (such as number of previous *P. falciparum* clinical episodes), geographical and temporal information (such as distance to wells and drug treatment period). There were 4,357 person-trimesters with at least one PFA out of a total of 23,832 person-trimesters from the 726 participating individuals. From hereon, we define an event as one person-trimester irrespective of the occurrence or not of a *P. falciparum* clinical episode and a positive event as a person-trimester with at least one PFA.

**Table 1:** List of explanatory categorical variables.

Categorical (nominal) Variables	No of levels
House	36
Independent Family	12
Sex	2
Haemoglobin Type (AA,AS,SS,AC,SC)	5
ABO blood group	4
G6PD Haplotype (on 4 SNPs: G6PD-376*, G6PD-202*, G6PD-968* and G6PD-542*)	11
PMI	2
POI	2
Birth during the project	2
**Categorical (ordered) Variables**	**No of levels**
Drug treatment period	4
Year	19
Trimester	4
ABO-261*: rs8176719	3
ABO-297*: rs8176720	3
ABO-467*: rs1053878	3
ABO-526*: rs7853989	3
ABO-771*: rs8176745	3
Alpha globin-3.7deletion	3
G6PD-202*: rs1050828	3
G6PD-376*: rs1050829	3
G6PD-542*: rs5030872	3
G6PD-968*: rs76723693	3

Note.-G6PD = Glucose-6-phosphate dehydrogenase, PMI = *P. malariae* infection, POI = *P. ovale* infection.

* Position on the amino-acid sequence.

**Table 2:** List of explanatory continuous variables.

Continuous Variables	Mean	Median	Min	Max
Age (year)	23.14	17.06	0	97.88
Mean genetic relatedness (Pedigree-based)	0.012	0.012	0.001	0.028
Mean genetic relatedness IBD-based)	0.008	0.008	0.002	0.025
Nb. of total PMI	4.1	1	0	44
Time since first PMI (year)	6.67	5.95	0	18.51
Nb. of total POI	1.33	0	0	11
Time since first POI (year)	6.2	5.55	0	18.51
Exposure (number of days present in the village) per trimester	81.65	91	1	92
Distance to animal enclosure (meters)	322	271	1	765
Distance to toilets (meters)	326	280	1	774
Distance to house's tree (meters)	344	311	1	759
Distance to wells (meters)	365	453	17	719
Distance to all (animals, toilets, house's tree, wells) together (meters)	329	288	1	774
Nb. of previous PFA	9.69	2	0	97

Note.Mean, median, minimum and maximum of the data are from the 726 participant individuals- IBD-Identity by Descent; PMI = *P. malariae* infection; POI = *P. ovale* infection; PFA = *P. falciparum* clinical episodes.

### Identification of Major Parameters

**Figure 1:**
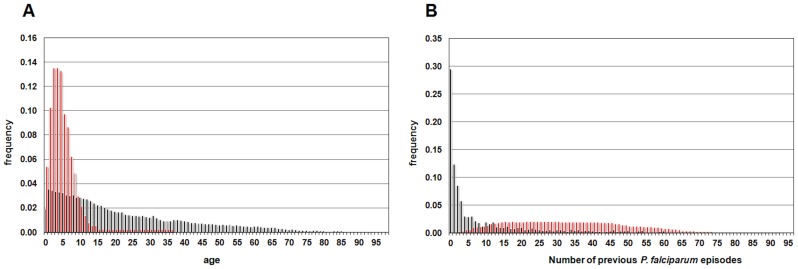
Histogram of the major variables. Histogram of age (A) and number of previous *P. falciparum* episodes (B), in the rules defining high risk of *P. falciparum* episodes produced by “DielmoAll” analysis (red boxes) and in the entire dataset of Dielmo village (black boxes).

In the HyperCube® analysis with all the variables, which was named “DielmoAll”, the 91 validated rules covered 3,521 events with “at least one PFA during the trimester” out of the 4,357 total number of events with at least one PFA (80.9%). The two major variables, which were present in more than 50% of the rules, were “Number of previous PFA, and “Age” (Table 3). The two variables explained 3,113 events from 259 individuals and 3,174 events from 262 individuals out of the 3,521 positive events, respectively. Interestingly, the number of rules using either “Age” (27 rules) or “Number of previous PFA” (37 rules) was significantly increased compared to those using either both (23 rules) or neither (4 rules) (*P*<0.001, Fisher’s exact test), suggesting that the two variables independently explained the risk of PFA in this village. Only 4 out of 91 rules used neither “Age” nor “Number of previous PFA”. Notably, our results suggest that young “Age” (Figure 1A), and high “Number of previous PFA” (Figure 1B) variables play major roles in explaining most of the susceptibility to PFA in this cohort. Whereas the first one is a well-known risk factor in endemic areas, the second has been hitherto considered as leading to protection, not increasing susceptibility.

**Table 3:** Data summary of the three analyses of Dielmo village.

Name of theanalysis	DielmoAll	DielmoNbprPFA	DielmoAge
Excludedvariable	None	NbprPFA*	Age
Nb of validatedrules	91	79	62
Coverage (%)**	3521(80.9)	3118(71.6)	2612(59.9)
Nb of rules withNbprPFA	60		62
Nb of rules withAge	50	79	
Nb of rules withYear	38	45	44

* Number of previous *P. falciparum* episodes.

** The coverage is the number (percentage) of positive events explained by the analysis.

To clarify the relationship between “Age” and “Number of previous PFA” defining susceptibility to PFA, we performed further analyses, excluding each major variable in turn. These analyses were named “DielmoNbprPFA” and “DielmoAge”. After excluding “Number of previous PFA” from the analysis, we obtained 79 validated rules all of which included the “Age” variable and explained 71.6% of all events with at least one PFA (Table 3). This result confirmed that the “Age” variable plays a major role in explaining the risk of *P. falciparum* clinical episodes. It also showed that in the absence of “Number of previous PFA”, the major variable “Age” will partially replace the explanatory power of this excluded variable. When excluding “Age” and the highly correlated variable “Birth in the project” from the analysis, 62 rules were validated covering 59.9% of the events with at least one PFA (Table 3). Thus, exclusion of the “Age” variable from the analysis induced a large loss of total number of rules and coverage, confirming the major influence of “Age” on PFA during each trimester (Table 3). Furthermore, all the 62 rules included the “Number of previous PFA” variable. These results showed that a younger “Age” and a high “Number of previous PFA” not only play major roles in explaining the risk of *P. falciparum* episodes but both of them partially replace the other when excluded.

### “Age” and “Number of Previous PFA” Explain Different Trimester-events

The above results gave the same weight to each rule. As these rules greatly differed in the number of events they contained, this could confound the interpretation of the results. To focus directly on the effect of the variables of interest on the number of events, we compared positive events (events with at least one PFA) included in at least one rule from “DielmoAll” analysis and “DielmoNbprPFA” analyses. The events lost after excluding “Number of previous PFA” from the analysis (DielmoNbprPFA) represented the events where the risk of PFA was essentially explained by “Number of previous PFA” and which could not be replaced by other variables including “Age”. The role of the “Age” variable was studied similarly. Each of these two variables defined events and individuals with different characteristics: 486 events from 148 villagers explained in the analysis with all the explanatory variables were lost after excluding “Number of previous PFA” variable. Similarly, 997 events from 275 villagers explained in the analysis with all the explanatory variables were lost after excluding the “Age” variable. The events lost after excluding “Number of previous PFA” had a significantly higher number of previous *P. falciparum* clinical episodes compared to events lost after excluding “Age” (mean = 25.8±0.9 and mean = 7.0±0.4, respectively, *P*<0.0001 Student’s t test) and were associated with a significantly higher age (mean = 13.24±0.40 year and mean = 3.45±0.11year, respectively, *P*<0.0001 Student’s t test). While susceptibility to clinical malaria in young children was explained by “Age”, the susceptibility in late adolescence was explained by “Number of previous PFA”. Using only children born during the 19-year survey did not modify these results (data not shown). Thus, the two variables, “Age” and “Number of previous PFA”, increased risk to PFA in different events.

### Validation in a Second Cohort

To exclude the possibility that these results were specific to the Dielmo cohort, we repeated these analyses in a second longitudinal cohort, Ndiop. These two adjacent villages differ in transmission intensity: holoendemic in Dielmo, and mesoendemic in Ndiop. Such differences are due to the fact that Dielmo village is close to a stream where mosquitoes breed all year in contrast to Ndiop village, which is situated in savannah and therefore only exposed to mosquitoes in the rainy season. The four major variables found by HyperCube® were “Semester”, “Age”, “Number of previous PFA” and “Year”. As found in Dielmo, in the analysis with all the variables (“NdiopAll” analysis), the number of rules using either “Age” (26 rules) or “Number of previous PFA” (23 rules) was significantly increased compared to those using either both (8 rules) or neither (3 rules) of these two variables (*P*<0.001, Fisher’s exact test, Table 4). After excluding “Age” from the analysis (“NdiopAge” analysis), the number of rules containing “Number of previous PFA” increased significantly (40 rules out of the 50 validated rules) compared to that of the analysis with all the variables (“NdiopAll” analysis, 31 rules out of 60 validated, *P*<0.003, Fisher’s exact test, Table 4). Similar results were obtained with the number of rules containing “Age”, 34 rules using “Age” out of 60 validated rules in the analysis without exclusion (“NdiopAll” analysis) compared to 47 rules out of 57 validated rules in the analysis excluding “Number of previous PFA” (“NdiopNbprPFA” analysis); (*P*<0.003, Fisher exact test, Table 4). Thus the results from Ndiop were similar to those found in Dielmo.

**Table 4:** Data summary of the five analyses of Ndiop village.

Name of the analysis	NdiopAll	NdiopNbprPFA	NdiopAge	NdiopYear	NdiopSemester
Excluded variable	None	NbprPFA*	Age	Year	Semester
Nb of validated rules	60	57	50	44	24
coverage (%)**	3998 (82.4)	3710 (76.5)	3245 (67.5)	3714(76.6)	2049 (42.2)
Nb of rules with NbprPFA	31	–	40	18	10
Nb of rules with Age	34	47	–	28	13
Nb of rules with Year	37	35	33	–	20
Nb of rules with Semester	53	53	46	43	–
Nb of rules with TimeNdiop/Semester	12	9	11	17	24

* The number of previous *P. falciparum* episodes.

** The coverage is the number (percentage) of positive events explained by the analysis.

### Validation by Classical Statistical Tools

**Figure 2:**
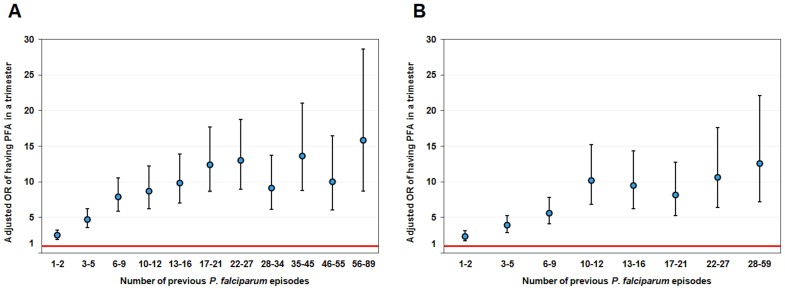
Odds ratio of having at least one *P. falciparum* clinical episode during the trimester. These odds ratios depend on the number of previous *P. falciparum* clinical episodes in either Dielmo village (A) or Ndiop village (B). Odds ratios were obtained by taking the exponential of the beta coefficients.

In order to validate the importance of number of previous PFA and assure that no confounding effect explained this increased risk of PFA resulting from a high number of previous PFA, we tested the effect of four variables on the risk of PFA during subsequent trimester using a generalized linear mixed model (GLMM) regression analysis. In contrast to HyperCube® data mining approaches, in the model-based analyses, we studied only individuals born in the study. These variables were “Age” and number of days in the village as continuous variables, drug treatment period and “Number of previous PFA”, as categorical variables. As shown in Figure 2A and Table 5 (and model residuals in Figure S1), the cumulative number of previous PFA increases significantly the risk of PFA in the subsequent trimester (*P*<10^−16^; likelihood ratio test between the model with NbprPFA and that without). A similar analysis was performed in Ndiop village but using semester instead of number of days in the village, because of the lower, seasonal malaria transmission. The results were similar to those found in Dielmo (Figure 2B and Table 6 and model residuals in Figure S2).

**Table 5:** Risk factors affecting clinical *P. falciparum* episodes in Dielmo village (model residuals shown in Figure S1).

Fixed effects	Estimate	Standard Error	z value	p-value
Intercept	−1.39	0.36	−3.91	9.22 10^−05^
NbprPFA_1–2*	0.90	0.14	6.56	5.46 10^−11^
NbprPFA_3–5	1.55	0.14	10.735	<2.0 10^−16^
NbprPFA_6–9	2.06	0.15	13.84	<2.0 10^−16^
NbprPFA_10–12	2.16	0.17	12.54	<2.0 10^−16^
NbprPFA_13–16	2.29	0.18	12.99	<2.0 10^−16^
NbprPFA_17–21	2.52	0.18	13.84	<2.0 10^−16^
NbprPFA_22–27	2.56	0.19	13.59	<2.0 10^−16^
NbprPFA_28–34	2.21	0.21	10.77	<2.0 10^−16^
NbprPFA_35–45	2.61	0.22	11.74	<2.0 10^−16^
NbprPFA_46–55	2.30	0.25	9.05	<2.0 10^−16^
NbprPFA_56–89	2.76	0.30	9.09	<2.0 10^−16^
Age**	−0.33	0.02	−17.39	<2.0 10^−16^
Days of presence	0.01	0.004	3.15	1.62 10^−−03^
Drug period 2	0.25	0.14	1.835	0.07
Drug period 3	−0.62	0.16	−3.79	1.51 10^−04^
Drug period 4	−0.88	0.17	−5.09	3.56 10^−07^

Random effects: Std. Dev._individuals_ = 0.58 (n = 285); Std. Dev._house_ = 0.00 (n = 32).

* The numbers after NbprPFA (Number of previous *P. falciparum* clinical episodes) give the range of the number of previous *P. falciparum* clinical episodes.

** No significant interaction between Age and Drug period.

**Table 6:** Risk factors affecting clinical *P. falciparum* episodes in Ndiop village (model residuals shown in Figure S2).

Fixed effects	Estimate	Standard Error	z value	p-value
Intercept	−2.77	0.28	−9.70	<2.0 10^−16^
NbprPFA_1–2*	0.85	0.15	5.79	7.04 10^−09^
NbprPFA_3–5	1.36	0.15	9.01	<2.0 10^−16^
NbprPFA_6–9	1.73	0.17	10.34	<2.0 10^−16^
NbprPFA_10–12	2.32	0.20	11.39	<2.0 10^−16^
NbprPFA_13–16	2.24	0.21	10.56	<2.0 10^−16^
NbprPFA_17–21	2.10	0.23	9.31	<2.0 10^−16^
NbprPFA_22–27	2.36	0.26	9.17	<2.0 10^−16^
NbprPFA_28–59	2.53	0.29	8.84	<2.0 10^−16^
Age**	−0.11	0.02	−4.55	5.30 10^−06^
Semester 2	3.08	0.09	33.55	<2.0 10^−16^
Drug period 2	−0.27	0.27	−1.00	0.32
Drug period 3	−0.95	0.29	−3.29	9.93 10^−04^
Drug period 4	−2.37	0.30	−7.92	2.34 10^−15^

Random effects: Std.Dev._individuals_ = 0.13 (n = 259); Std.Dev._house_ = 0.01 (n = 26).

* The numbers after NbprPFA (Number of previous *P. falciparum* clinical episodes) give the range of the number of previous *P. falciparum* clinical episodes.

** No significant interaction between Age and Drug period.

### Influence of “Age” and Minor Variables on the Effect of “Number of Previous PFA” on Risk of Subsequent PFA

**Figure 3:**
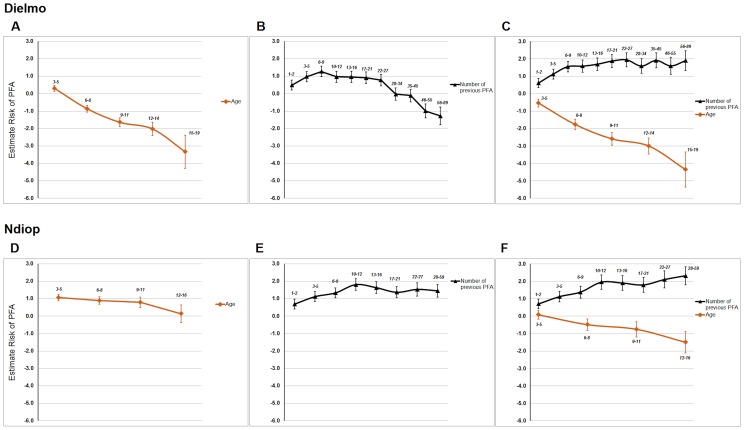
Estimated effects (beta coefficients) of having at least one *P. falciparum* clinical episode during the trimester in different models. These estimated effects were calculated in either Dielmo village (A to C) or Ndiop village (D to F) for “Age” alone (A and D), “Number of previous PFA” alone (B and E), and both variables (C and F).

As the “Age” variable is positively related to the “Number of previous PFA” variable, a confounding effect of “Age” on “Number of previous PFA” could occur. One such confounding effect would arise if the relationship of “Age” variable and risk of PFA was not linear but curved. If this were to be the case, fitting “Age” variable as a linear covariate would be wrongly adjusted by adding “Number of previous PFA” variable to the model. To exclude such a possible confounding effect of “Age” on the “Number of previous PFA”, their effects on the risk of subsequent PFA were tested together and separately in the model using GLMM and using only individuals born in the study (Tables S1–S3 for Dielmo and Tables S4–S6 for Ndiop and model residuals in Figure S3). After exclusion of “Age” from analysis of Dielmo village (Table S2, Figure 3B), all categories with less than 27 previous PFA increase the risk of subsequent PFA. Only categories with more than 46 previous PFA decrease the risk of subsequent PFA, probably due to the “Age” effect. A similar analysis in Ndiop village after excluding “Age” (Table S5, Figure 3E) showed that all categories of “Number of previous PFA” increase the risk of subsequent PFA. In this last village after exclusion of “Number of previous PFA” from the analysis (Table S6), “Age” surprisingly increased risk of PFA. In conclusion, these results excluded a major confounding effect of “Age” on “Number of previous PFA” even if both variables are positively correlated. These previous models hypothesized a linear relationship of “Age”, when used as a continuous variable, with the risk of subsequent PFA. We confirmed this linear relationship of “Age” by models using “Age” as a categorical variable (Figure 3A–C, and Table S7 and S8 for Dielmo; Figure 3D–F, and Table S9 and S10 for Ndiop). These analyses in Ndiop village explained the surprising result of “Age” increasing the risk of subsequent PFA after exclusion of “Number of previous PFA” (Figure 3D). Very young children of less than 3 years old were protected from the risk of PFA. After that period, the risk of PFA greatly increased and was stable until eleven years of age. The risk of PFA began to decrease after this age. When both “Age” and “Number of previous PFA” were included in the analyses (Figure 3C Dielmo, Figure 3F Ndiop), their effects were the same as when “Age” was used as a continuous variable. All results from Figure 3 and Tables S7–S10 were against a confounding effect of “Age” on “Number of previous PFA”. They were also compatible with the hypothesis that “Number of previous PFA” affects risk of subsequent PFA independently of its relationship with “Age”, even if both variables are positively related. This last hypothesis was strongly supported by results obtained after stratification according to age (Table S11 and S12, Figure S4 and S5), even if the effect of “Number of previous PFA” on the risk of subsequent PFA decreased for the oldest children in both villages and was lower in Ndiop village than in Dielmo village. This effect of the “Number of previous PFA” was delayed in Ndiop village, likely because of the lower transmission intensity (Summary S1).

Furthermore, to exclude the possibility that another confounding variable affects this result, we included important minor variables identified by the HyperCube® analysis in the GLMM regression analysis using only individuals born in the study; minor variables were defined either as those variables being present in 25% to 50% of the rules in the DielmoAll analysis or being significantly associated with rules containing “Number of previous PFA” variables. Three such minor variables were identified: “Number of total *P. malariae* infections”, and “Year” variables, which are present respectively in 39 and 38 out of the 91 rules, and “Number of total *P. ovale* infections” variable, which was significantly associated with “Number of previous PFA” variable, which was in 14 out of the 15 rules containing “Number of total *P. ovale* infections” (Fisher’s exact test p = 0.016). We had already taken into account the “Year” variable in the GLMM regression analysis by using another highly correlated variable, “drug treatment period”. Addition of “Number of total *P. malariae* infections” and “Number of total *P. ovale* infections” variables in the GLMM regression analysis did not affect the increased risk of PFA associated with a high number of previous PFA (data not shown). Also, previous results from the Dielmo project and elsewhere have shown that individuals with the sickle cell trait have a decreased risk of *P. falciparum* infections [11]. In the DielmoAll analysis, haemoglobin genotype was present in only 6 rules, five of which did not include the “Number of previous PFA” variable, excluding a confounding role of the sickle cell trait (Fisher’s exact test p = 0.016). In conclusion, we were unable to show that any of the 35 variables had a confounding effect explaining that of “Number of previous PFA”.

### Influence of Environmental Factors on the Effect of “Number of Previous PFA” on Risk of Subsequent PFA

We cannot exclude the possibility that another variable not present among the other 35 variables is confounding. One possibility is that an environmental factor is responsible for the clustering of *P. falciparum* clinical episodes in a part of Dielmo village. To test that hypothesis, we defined two groups of individuals: those present in rules with “Number of previous PFA” that continue to be susceptible to PFA and those present in rules with “Number of previous PFA” who became resistant to PFA. For each individual of these two groups, the localization inside Dielmo village was defined (see Material and Methods for details). Figure S6 shows the localization of individuals of these two groups, respectively 47 susceptible and 41 resistant individuals. Although the pattern of localization of these two groups of individuals is similar inside Dielmo village, there is significant clustering (Moran’s index = 0.026 P = 0.002), largely due to a group of individuals in the most northern part of the village. We also performed a similar analysis with individuals present in rules with “Age”. There were 46 susceptible and 33 resistant individuals. Results are shown in Figure S7. The pattern of localization of these two groups of individuals present in rules with “Age” is similar, with no evidence of clustering (Moran’s index = 0.000104 P = 0.63). Thus, there is some evidence of geographical clustering of individuals with a high number of previous PFA, but who become resistant to *P. falciparum* episodes. Further investigation of this clustering will need to be performed.

## Discussion

The goal of this paper was to search for new risk factors of susceptibility to *P. falciparum* clinical episodes in the light of the disparity in numbers of clinical episodes among children of the same age in a well-documented longitudinal cohort [8]. We used a reverse engineering approach to identify the most biologically relevant variables among the 36 variables registered during the following 19-year study. Using this hypothesis-free approach, we implemented an exhaustive search of our extensive malaria data set to generate groups of risk factors that contribute to susceptibility to *P. falciparum* clinical episodes.

The results confirm the major role of age in determining susceptibility to PFA [6]. The other major risk variable was the individual’s history of *P. falciparum* episodes (Tables 3 and 4). The “Age” and “Number of previous PFA” variables were risk factors for different groups of individuals. For the most part, the events defined by rules using the “Number of previous PFA” variable had different characteristics to those defined in rules using the “Age” variable, notably an older age and a higher number of previous PFA. To definitely validate that “Age” variable had no confounding effect on “Number of previous PFA” variable, we performed different classical statistical analyses on individuals born during the study to exclude bias induced by partial knowledge of an individual’s history. These analyses and others confirmed that no confounding variable for “Number of previous PFA” was detected among the other 35 variables, although there was some clustering inside the village, albeit of individuals with a high number of previous PFA who became resistant.

**Figure 4:**
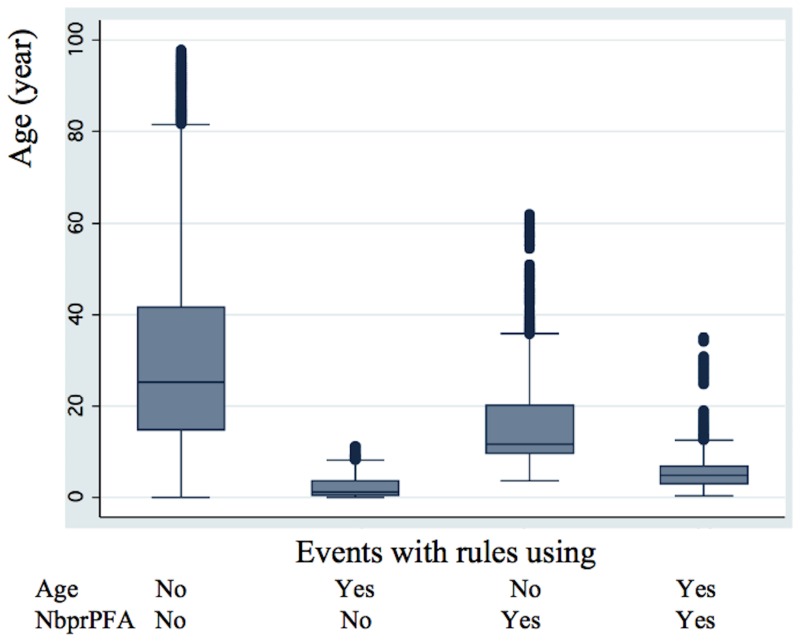
Age distribution of events present in rules using either “Age” or “Number of previous PFA”, both or neither in “DielmoAll” analysis. Events are divided into four groups depending on their presence/absence within the two rules, one using “Age” and the other “Number of previous PFA” variables. After verifying by a one-way ANOVA that the four groups have different mean ages (F (3,23770) = 3350.86; *P*<0.0001), the Scheffe test shows that each of the four groups has a mean age significantly different from the others (*P*<0.001).

The finding that a high value for the variable “ Number of previous *P. falciparum* episodes” is a risk factor for subsequent clinical episodes in certain individuals contributes to the observed disparity in numbers of clinical episodes among children of the same age in the first cohort (Dielmo village). A high value for “Number of previous PFA” variable was also observed to be a risk factor in a second cohort (Ndiop village). This result is in contrast to the effect that a history of exposure is commonly believed to have: a high number of previous *P. falciparum* episodes leads to the induction of clinical immunity [1]. Importantly, however, this risk factor is only pertinent for some individuals, leading to a delayed acquisition of clinical immunity compared to the rest of the child population, where peak incidence occurs at 3–4 years of age in Dielmo and 8–9 years in Ndiop. This delay in acquisition of clinical immunity in the novel susceptibility group is well illustrated in Figure 4 (for Dielmo) and Figure S8 (for Ndiop) that show the age distributions of risk associated with rules containing “Age” and those containing “Number of previous PFA”. Increased susceptibility of specific sub-groups has previously been noted for pregnant women, notably primigravidae, likely as a result of the associated physiological changes during pregnancy and the deleterious effects of placental malaria [16]. This cannot account for the susceptibility group observed here, suggesting that additional factors are interfering with the development of clinical immunity in this specific group. The delayed development of clinical immunity in this group is likely to have been exacerbated by drug treatment [17], [18], because of being treated too rapidly to allow an appropriate immune response. This group may also possibly have a poorer response to vaccines, being less capable of developing acquired immunity [19], [20]. They will also likely disproportionally contribute to the disease burden -and possibly transmission- during the first phases of malaria eradication when the force of infection drops, as has been recently highlighted [21]: this group potentially represents one of the “Red Queen hurdles” that will complicate malaria eradication in Africa [21]. Our results emphasise the importance of heterogeneity of risk with different factors coming into play in individuals exposed to the same transmission intensity [22], [23], resulting in a more complex relationship between age and development of acquired immunity [24], [25]. Identifying biomarkers that are proxies for this high-risk group would be a major step in enabling implementation of specific prevention measures and allowing adequate study population stratification.

Our results strongly suggest that both age and the number of previous PFA have to be taken into account when evaluating clinical trials. The occurrence of differently susceptible sub-groups of children poses an immediate problem for population stratification in clinical trials. Whilst age can be incorporated to stratify the study population, the number of previous PFA requires long-term studies beyond that which is reasonable for the size of cohorts necessary for clinical trials. Identifying markers that are proxies for this high-risk group would be a major step in enabling additional study population stratification. Interestingly, a previous study in the same cohort on the delay of reappearance of *P. falciparum* after radical treatment found that re-parasitization depended not only on age and haemoglobin genotype, but also on the pre-treatment parasitemia [26]. We found that age and number of previous PFA affect *P. falciparum* density during asymptomatic infections (Table S13): parasitaemia decreased with age but increased with number of previous PFA. This clearly points to the impaired development of an anti-parasite immune response. Asymptomatic parasite density may, therefore, be useful in estimating the extent to which children belong to the highly susceptible risk group.

The present study gives little insight into the underlying biological reasons for this new risk factor. Further studies will be performed, with particular focus on the specific associations of the minor variables with “Number of previous PFA”, notably the effects of *P. ovale* and *P. malariae* and measure of immunoglobin titres, IgG1&3, known to be crucial for effective acquired immunity [27]. We are also searching for hotspots of Malaria transmission, which might contribute to the effect of “Number of previous PFA” [28].

In conclusion, clinical immunity to *P. falciparum* does not *de facto* occur with increasing history of exposure to infection. On the contrary, it appears that certain individuals enter into a vicious cycle of repeated clinical episodes, inducing a delay in the appearance of clinical immunity against *P. falciparum* episodes. Whilst the reasons for this are currently under investigation, it is clear that such a risk factor needs to be taken into account not only for vaccine trials but more broadly when assessing any intervention method.

## Materials and Methods

### Participants

Between 1990 and 2008, a longitudinal study involving the inhabitants of the villages of Dielmo and Ndiop (1994–2008), Senegal, was carried out to identify all episodes of fever. The study design included daily medical surveillance with systematic blood testing of individuals with fever and examination of 200 oil-immersion fields on a thick blood film for malaria parasites (about 0·25 µL of blood).

The Dielmo village is situated in a Sudan-savannah region of central Senegal, on the marshy bank of a small permanent stream, where anopheline mosquitoes breed all year round [6], [29]. Malaria transmission is intense and perennial, with a mean 258 infected bites per person per year during 1990–2006 in Dielmo [30] and ten-fold lower in Ndiop [31].

Written informed consent was obtained from all participants in our study or the guardians of children younger than 15 years. Our project was initially approved by the Ministry of Health of Senegal and the assembled village population. Approval was then renewed on a yearly basis. Audits were done regularly by the National Ethics Committee of Senegal and *ad hoc* committees of the Ministry of Health, the Pasteur Institute (Dakar, Senegal), and the Institut de Recherche pour le Développement (Marseille, France).

### Procedures

We gave each individual a unique identification code for our project and prepared a file that contained a photograph, details of family ties, occupation, and precise place of residence on detailed maps of each household with the location of each bedroom. We visited all households daily, and collected nominative information 6 days a week (i.e. excluding Sunday) at home on the presence or absence in the village of each individual we had enrolled, their location when absent, and the presence of fever or other symptoms. We systematically recorded body temperature at home three times a week (every second day) in children younger than 5 years, and in older children and adults in cases of suspected fever or fever-related symptoms. In cases of fever or other symptoms, blood testing was done at our dispensary by finger prick, and we provided detailed medical examination and specific treatment. The dispensary created for our project was open 24 h a day, 7 days a week to allow both active and passive case detection.

We treated parasitologically confirmed clinical malaria episodes according to national guidelines. From 1990 to 2008, four different drug regimens were implemented: *Quinine* from 1990 to 1994, *Chloroquine* from 1995 to 2003, *Fansidar* from 2004 to mid-2006 and *Artemisinin-based combination therapy (ACT)* from mid-2006 to 2008. We measured treatment efficacy with daily clinical surveillance of patients and with at least one control of parasitaemia between day 7 and day 35 after fever resolved.

The outcome of interest is a *P. falciparum* malaria clinical episode (PFA). PFA was defined as a clinical presentation with measured fever or fever-related symptoms associated with a *P. falciparum* parasite/leukocyte ratio higher than an age-dependent pyrogenic threshold previously identified in the patients from Dielmo [32]. The threshold was used because of high prevalence of asymptomatic infections in the population, as occurs in regions endemic for malaria. In Ndiop, a threshold of 0.3 parasites/leukocyte was used irrespective of age.

Some explanatory variables are time-dependent and were therefore evaluated for each trimester. These included current age, experience of exposure to other *Plasmodium* spp. (*P. ovale* and *P. malariae*). Other variables are individual-dependent including sex, geographical location (e.g. house), and genetic profiles. Experimental details are available in a previous paper [15]. The “Number of previous PFA” refers to the total number of PFA before the present trimester. All variables are summarized in Tables 1 and 2.

### HyperCube® Data Mining Algorithm

The HyperCube® technology is accessible as a web based software that requires no specific learning skills, though it requires a significant computing power provided through a SaaS architecture (Bussiness EffiScience, Paris, France). The program exhaustively searches for local over-densities of a class of the dependent variable in the n-dimensional space of the explanatory variables using a non-Euclidean and non-parametric approach. Each over-density defined a hypercube, a subspace of the n-dimensional space, under two main constraints: the “Lift”, the ratio of the prevalence of positive outcome events with at least one PFA/trimester within a rule over the prevalence in the entire population, which is equivalent to the relative risk (RR), the “Size”, the number of events included in the hypercube. For each hypercube, three steps were performed: First, the program defined the minimal number of variables necessary to explain an over-density satisfying constraints defined by the user; Second, its “Size” is maximized under the constraint of the “Lift” by using genetic algorithms; Third, statistical significance of each hypercube is tested by random permutation of the dependent variable. Each hypercube defines a rule, which is a combination of explanatory variables each of which is associated with either a range (for a continuous variable) or a modality (for a discrete variable). The program stops either when all the events of the learning dataset of the dependent variable have been explained (i.e. all the events are present in at least one rule) or when all the possible hypercubes have been studied or after a manual stop order. Before stopping, a set of minimized rules is obtained from the total number of rules using the following iterative process. In the first step, the rule explaining the most number of events is chosen and the events explained by this rule excluded from space. At each of the following steps, the rule explaining the maximal number of events is added and the events explained by this rule excluded. This iterative process is stopped when all the events explained by the total number of rules are explained by the set of minimized rules. The total number of rules and/or the minimized rules can be downloaded onto the local computer to perform further analysis. Further details are given in a previous paper [15].

### Description of the Analyses

Each analysis contains two phases using 2 randomly selected data sets: a Learning set (11,893 events), and a Validation set (11,939 events). Using the Learning set, HyperCube® generates a list of rules, each containing a specific combination of variables, which reflect local over-density of events with “at least one PFA during the trimester”, and which satisfy the two main user-defined constraints: the “Lift” and the “Size”. The “Lift” parameter is the minimum relative risk (RR) of the events with the “at least one PFA during the trimester” class present in the rule compared to the entire dataset from Dielmo village. The “Size” parameter is the minimum number of events explained by a rule. Each rule is a set of a limited number of quantitative and/or qualitative variables and their associated values. The “Complexity” parameter can be used to define the maximum number of variables in a rule. We used the same values for these 3 parameters in all the HyperCube® analyses: “Lift” (minimum relative risk) = 4, “Size” (minimum number of events explained by a rule) = 250, and “Complexity” (maximum number of variables in a rule) = 6. A reduced set of minimum number of rules containing all the individual events explained by the full list of rules is then generated. Such minimized rules are then used in the rest of the analysis. They are validated using the Validation set with 10^−80^ as the p-value cut-off to account for over-fitting. We defined as major variables those that are used more than 50% inside the set of rules. New HyperCube® analyses were performed after excluding one of the major variables to understand relationship amongst major variables. The first analysis containing all variables was named “DielmoAll” and the following ones by Dielmo plus the name of the excluded major variable.

### Statistical Analyses

The dependence of factors in two-way tables was tested using Fisher’s exact test. Two means (mean ± S.E. (number of events)) were compared by Student’s t test. Overall difference of more than two means was tested using ANOVA with pair-wise difference tested using Scheffe’s test. Differences were considered significant if the p-value was lower or equal to 0.05.

To validate the effects of number of previous PFA on the risk of PFA during the subsequent trimester, we used Generalized Linear Mixed Models (GLMM) using *pedigreemm* package in R free software [33]. This performs mixed models taking into account both non-independence among individuals due to their genetic relationship and repeated measures. Occurrence or not of PFA was modelled as a binary outcome, its “*logit*” transformation was used as the link function between E(PFA *| covariates*) and a linear combination of the four fixed covariates: “Age” (continuous), drug treatment period, “Number of previous PFA” and either Number of days of presence/trimester (Dielmo) or Semester (Ndiop) and two random covariates: individual and house. In the supplementary tables and results of Figure 3, drug treatment period was studied as a random covariate. Non-independence of individual effects was modelled by incorporating the kinship matrix between individuals as a matrix of variance-covariance between subject (see the technical note of the authors of *pedigreemm* R-package [33]; non-independence of repeated measures within individuals was modelled by allowing the “UNSTRUCTURED” covariance matrix (the default option) and then the empirical variance-covariance matrix was used. In contrast to HyperCube® data mining approach, we studied only individuals born in the study to exclude bias induced by partial knowledge of an individual’s history. In addition we excluded from analyses any observations of each trimester for which the individual concerned was not present for at least one third of the time (i.e. 30 days). When clinical *P. falciparum* episodes were coded as a quantitative trait, it was modelled as a Poisson outcome and the link function was the log function. For studying “Number of previous PFA” stratified according to age, we used the following protocol: age was divided into 5 groups, each defining 20% of the events; then, for each age group, “Number of previous PFA” was divided into 4 groups, again each defining 25% of the events within that age group. Odds ratios were obtained by taking the exponential of the beta coefficients.

### Localization of Individuals in the Dielmo Village

First, we identified events present in at least one rule containing “Number of previous PFA” from the “DielmoAll” analysis. These events will help to define a group of high risk individuals. High risk individuals are those who are present in more than 4 rules containing “Number of previous PFA”. For each high risk individual, the percentage of events with at least one PFA during the trimester was calculated after weighting each event by the number of rules in which it was present. A high risk individual was classified as “susceptible” if the weighted percentage of events with at least one PFA during the trimester was greater or equal to 72.74%. This threshold of 72.74% is identical to a risk ratio of 4, the threshold for generating rules with an over-density of events with “at least one PFA during the trimester” in all HyperCube® analyses. High risk individuals were “resistant” if this percentage was lower than 36.37% (risk ratio <2 in the HyperCube® analysis). These high risk individuals are not “resistant” in the classical sense of having a risk ratio less than one, but relatively so compared to all the high risk individuals. The number of susceptible, resistant, and unclassified individuals with known house localization were respectively 47, 41 and 271. Similar calculi were performed for events present in at least one rule containing “Age”. The number of susceptible, resistant, and unclassified individuals due to “Age” with known house localization were respectively 46, 33 and 270.

Spatial autocorrelation analyses were carried out in ArcGis and the methods used are explained in depth at the following website http://resources.arcgis.com/en/help/main/10.1/index.html. In brief, the Spatial Autocorrelation (Global Moran's I) tool measures spatial autocorrelation based on both feature locations and feature values simultaneously. Given a set of features and an associated attribute, it evaluates whether the pattern expressed is clustered, dispersed, or random. The tool calculates the Moran's I Index value and both a z-score and p-value to evaluate the significance of that Index. P-values are numerical approximations of the area under the curve for a known distribution, limited by the test statistic.

## Supporting Information

Figure S1 **Distribution of residuals from model described in** **Table 5** **.**
(TIF)

Figure S2 **Distribution of residuals from model described in** **Table 6** **.**
(TIF)

Figure S3 **Distribution of residuals from models described in Table S1 to S6.**
(TIF)

Figure S4 **Histogram of Number of PFA during a trimester for individuals born during the project and living in Dielmo village before ACT therapy.** A: Age <3 and NbprPFA <5; B: Age <3 and NbprPFA > = 5; C: 3< = Age <6 and NbprPFA <20; D: 3< = Age <6 and NbprPFA > = 20; E: 6< = Age <9 and NbprPFA <35; F: 6< = Age <9 and NbprPFA > = 35; G: Age > = 9 and NbprPFA <50; H: Age > = 9 and NbprPFA > = 50.
(TIF)

Figure S5 **Histogram of Number of PFA during a trimester for individuals born during the project and living in Ndiop village during the rainy season before ACT therapy.** A: Age <3 and NbprPFA <3; B: Age <3 and NbprPFA > = 3; C: 3< = Age <6 and NbprPFA <10; D: 3< = Age <6 and NbprPFA > = 10; E: 6< = Age <9 and NbprPFA <20; F: 6< = Age <9 and NbprPFA > = 20; G: Age > = 9 and NbprPFA <45; H: Age > = 9 and NbprPFA > = 45.
(TIF)

Figure S6 **Localization of susceptible (red circle) and resistant (green circle) individuals as defined by “Number of previous PFA” inside Dielmo village.**
(TIF)

Figure S7 **Localization of susceptible (red circle) and resistant (green circle) individuals as defined by “Age” inside Dielmo village.**
(TIF)

Figure S8 **Age distribution of events defined by rules using either “Age” or “Number of previous PFA”, or both or neither in Ndiop village.** After verifying by a one-way ANOVA that the four groups do not have identical mean ages (F(3,22510) = 1387.17; *P*<0.0001), the Scheffe test shows that each group has a mean age that is significantly different from the other (*P*<0.001).
(TIF)

Table S1 **Risk factors affecting clinical** ***P. falciparum*** **episodes in Dielmo village (All factors).**
(DOC)

Table S2 **Risk factors affecting clinical** ***P. falciparum*** **episodes in Dielmo village (Exclusion of Age).**
(DOC)

Table S3 **Risk factors affecting clinical** ***P. falciparum*** **episodes in Dielmo village (Exclusion of NbprPFA).**
(DOC)

Table S4 **Risk factors affecting clinical** ***P. falciparum*** **episodes in Ndiop village (All factors).**
(DOC)

Table S5 **Risk factors affecting clinical** ***P. falciparum*** **episodes in Ndiop village (Exclusion of Age).**
(DOC)

Table S6 **Risk factors affecting clinical** ***P. falciparum*** **episodes in Ndiop village (Exclusion of NbprPFA).**
(DOC)

Table S7 **Risk factors affecting clinical** ***P. falciparum*** **episodes in Dielmo village (All factors; Age analyzed as categories).**
(DOC)

Table S8 **Risk factors affecting clinical** ***P. falciparum*** **episodes in Dielmo village (Exclusion of NbprPFA; Age analyzed as categories).**
(DOC)

Table S9 **Risk factors affecting clinical** ***P. falciparum*** **episodes in Ndiop village (All factors; Age analyzed as categories).**
(DOC)

Table S10 **Risk factors affecting clinical** ***P. falciparum*** **episodes in Ndiop village (Exclusion of NbprPFA; Age analyzed as categories).**
(DOC)

Table S11 **Risk factors affecting clinical** ***P. falciparum*** **episodes stratified according to Age in Dielmo village.**
(DOC)

Table S12 **Risk factors affecting clinical** ***P. falciparum*** **episodes stratified according to Age in Ndiop village.**
(DOC)

Table S13 **Factors affecting maximal asymptomatic parasite density during a trimester.**
(DOC)

Summary S1 (DOC)
