# Supplementary figures and images for: High Number of Previous Plasmodium falciparum Clinical Episodes Increases Risk of Future Episodes in a Sub-Group of Individuals
Source: PLoS One. 2013 Feb 6;8(2):e55666. doi: 10.1371/journal.pone.0055666 (PMC3566008; doi:10.1371/journal.pone.0055666)

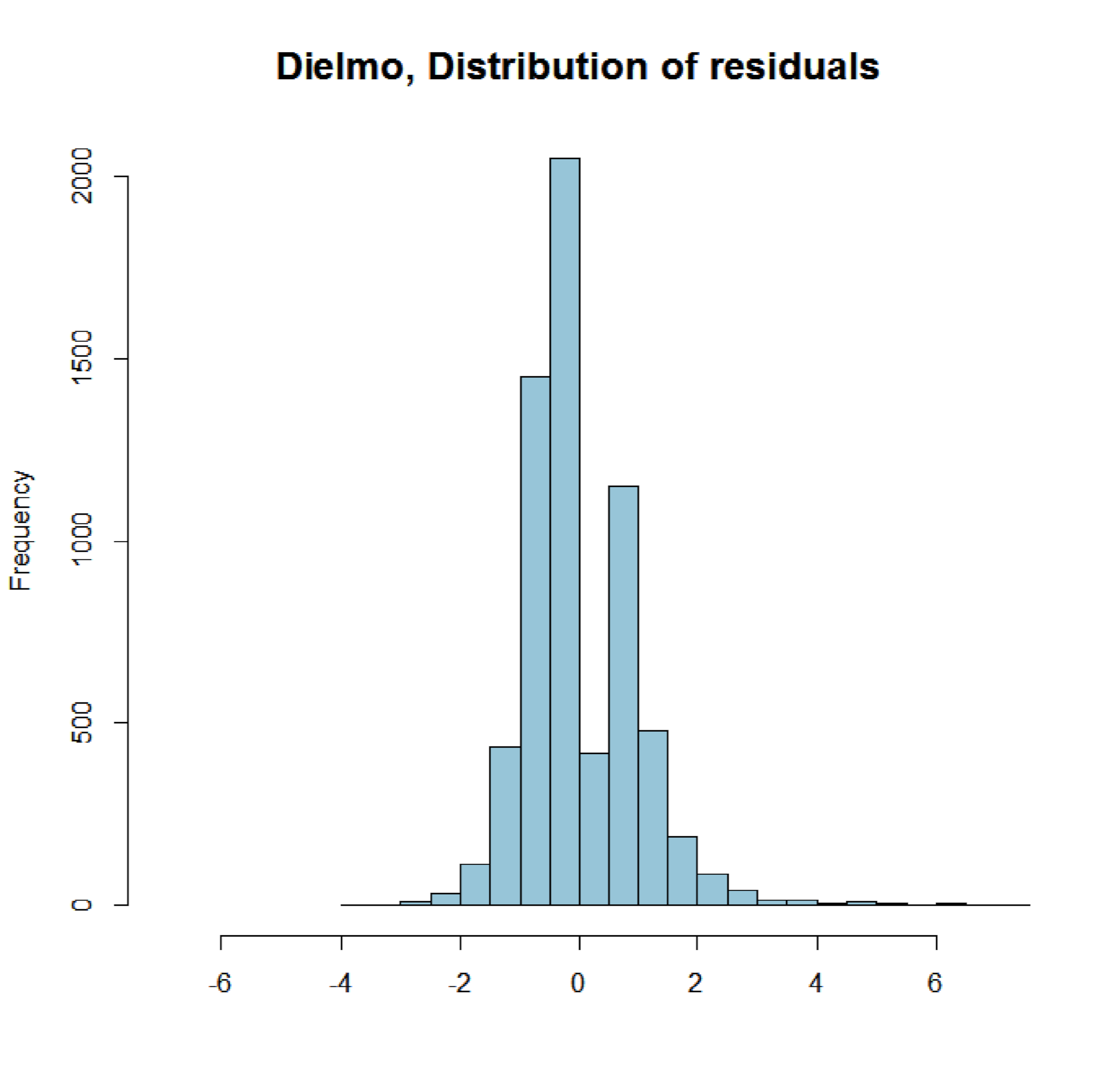

Supplement: Figure S1 — Distribution of residuals from model described in Table 5 . (TIF) [file pone.0055666.s001.tif]

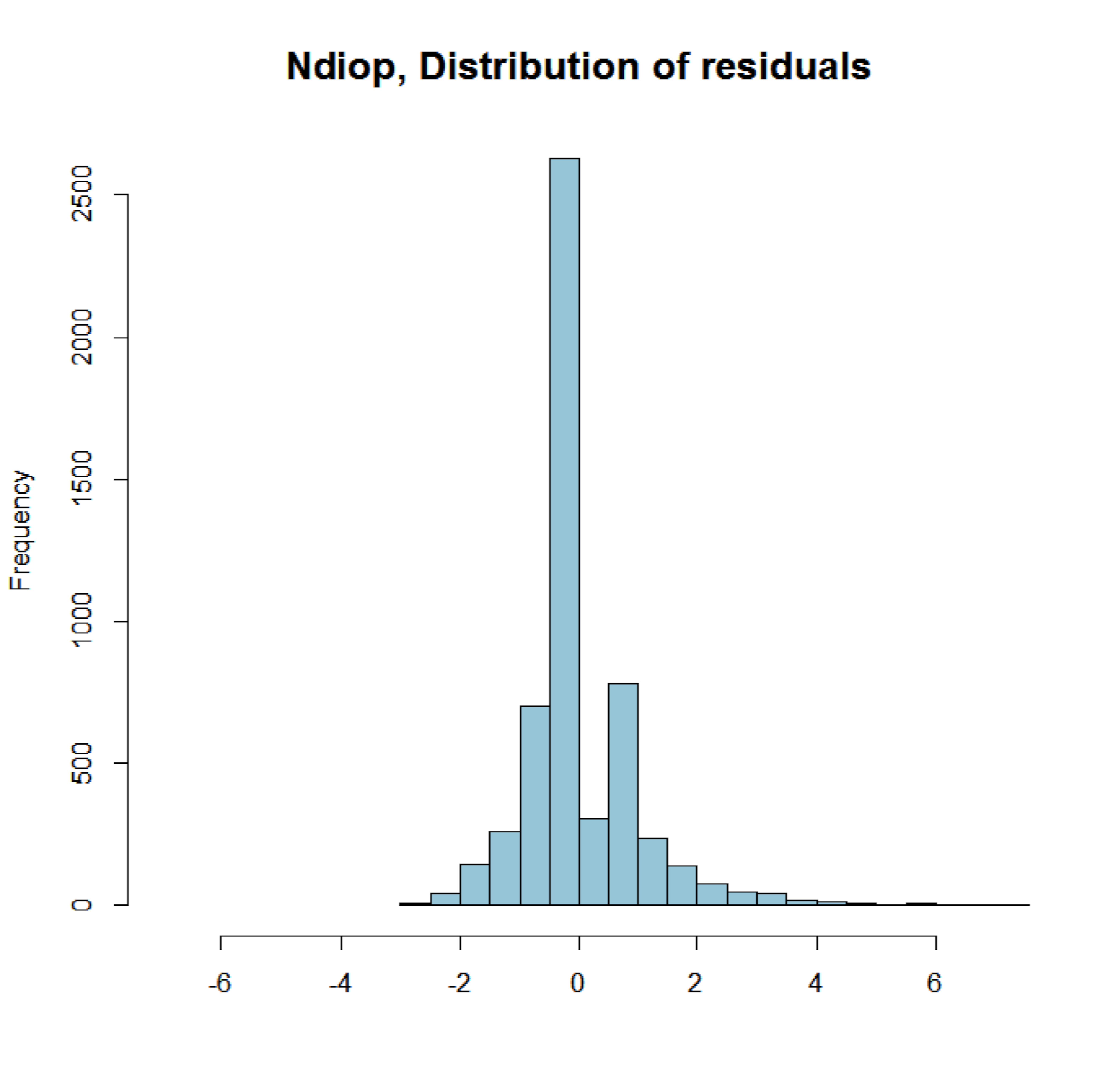

Supplement: Figure S2 — Distribution of residuals from model described in Table 6 . (TIF) [file pone.0055666.s002.tif]

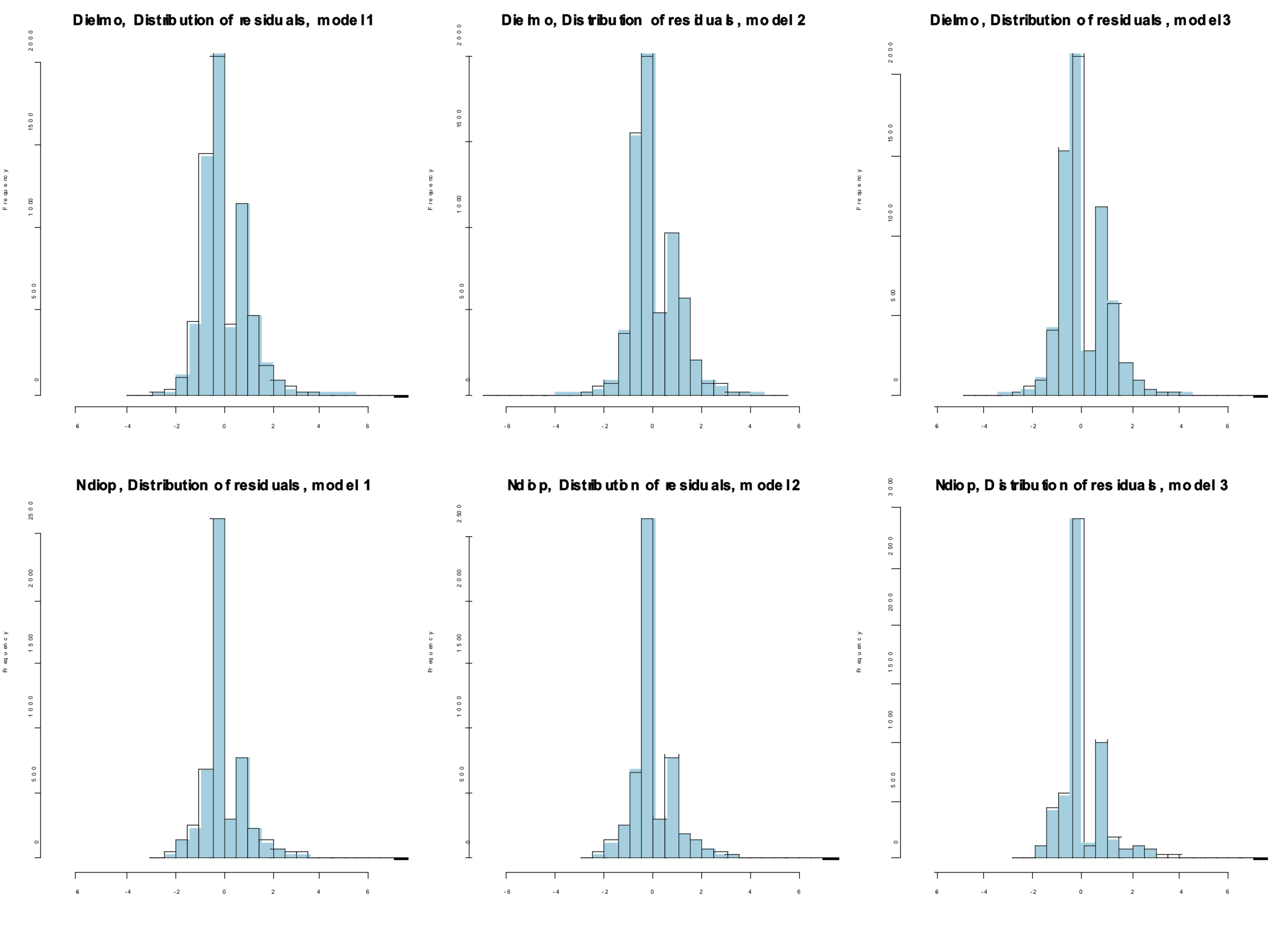

Supplement: Figure S3 — Distribution of residuals from models described in Table S1 to S6. (TIF) [file pone.0055666.s003.tif]

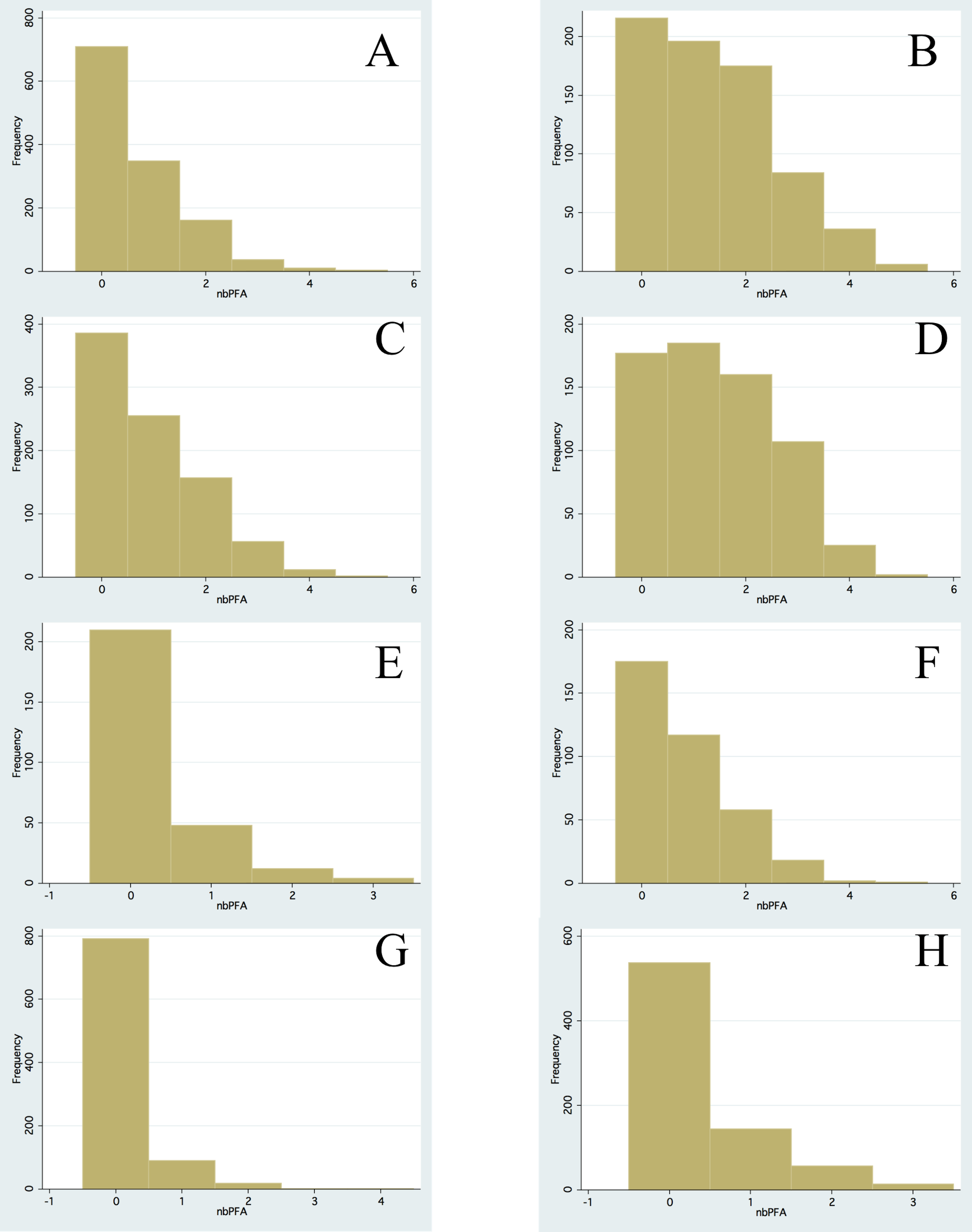

Supplement: Figure S4 — Histogram of Number of PFA during a trimester for individuals born during the project and living in Dielmo village before ACT therapy. A: Age <3 and NbprPFA <5; B: Age <3 and NbprPFA > = 5; C: 3< = Age <6 and NbprPFA <20; D: 3< = Age <6 and NbprPFA > = 20; E: 6< = Age <9 and NbprPFA <35; F: 6< = Age <9 and NbprPFA > = 35; G: Age > = 9 and NbprPFA <50; H: Age > = 9 and NbprPFA > = 50. (TIF) [file pone.0055666.s004.tif]

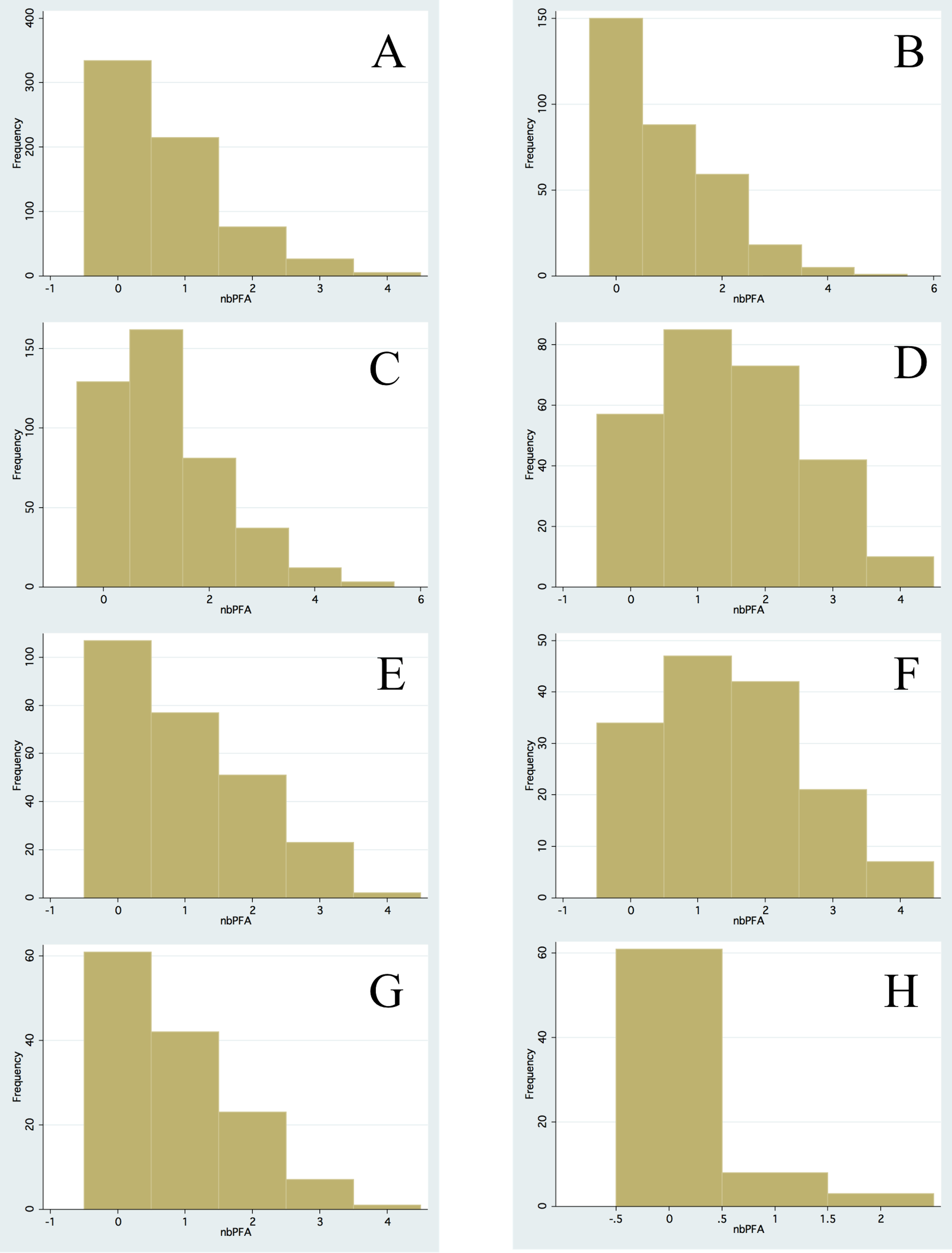

Supplement: Figure S5 — Histogram of Number of PFA during a trimester for individuals born during the project and living in Ndiop village during the rainy season before ACT therapy. A: Age <3 and NbprPFA <3; B: Age <3 and NbprPFA > = 3; C: 3< = Age <6 and NbprPFA <10; D: 3< = Age <6 and NbprPFA > = 10; E: 6< = Age <9 and NbprPFA <20; F: 6< = Age <9 and NbprPFA > = 20; G: Age > = 9 and NbprPFA <45; H: Age > = 9 and NbprPFA > = 45. (TIF) [file pone.0055666.s005.tif]

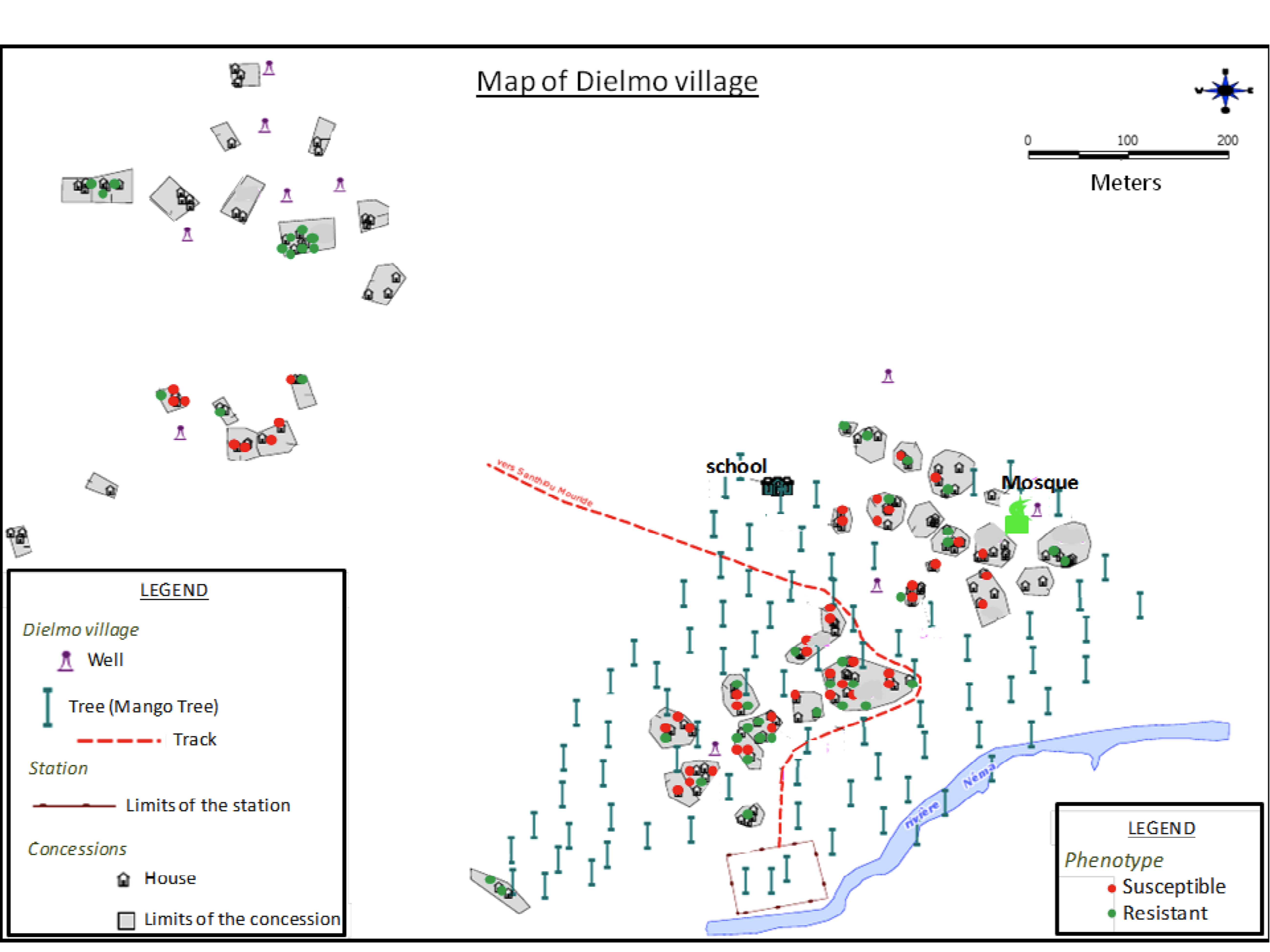

Supplement: Figure S6 — Localization of susceptible (red circle) and resistant (green circle) individuals as defined by “Number of previous PFA” inside Dielmo village. (TIF) [file pone.0055666.s006.tif]

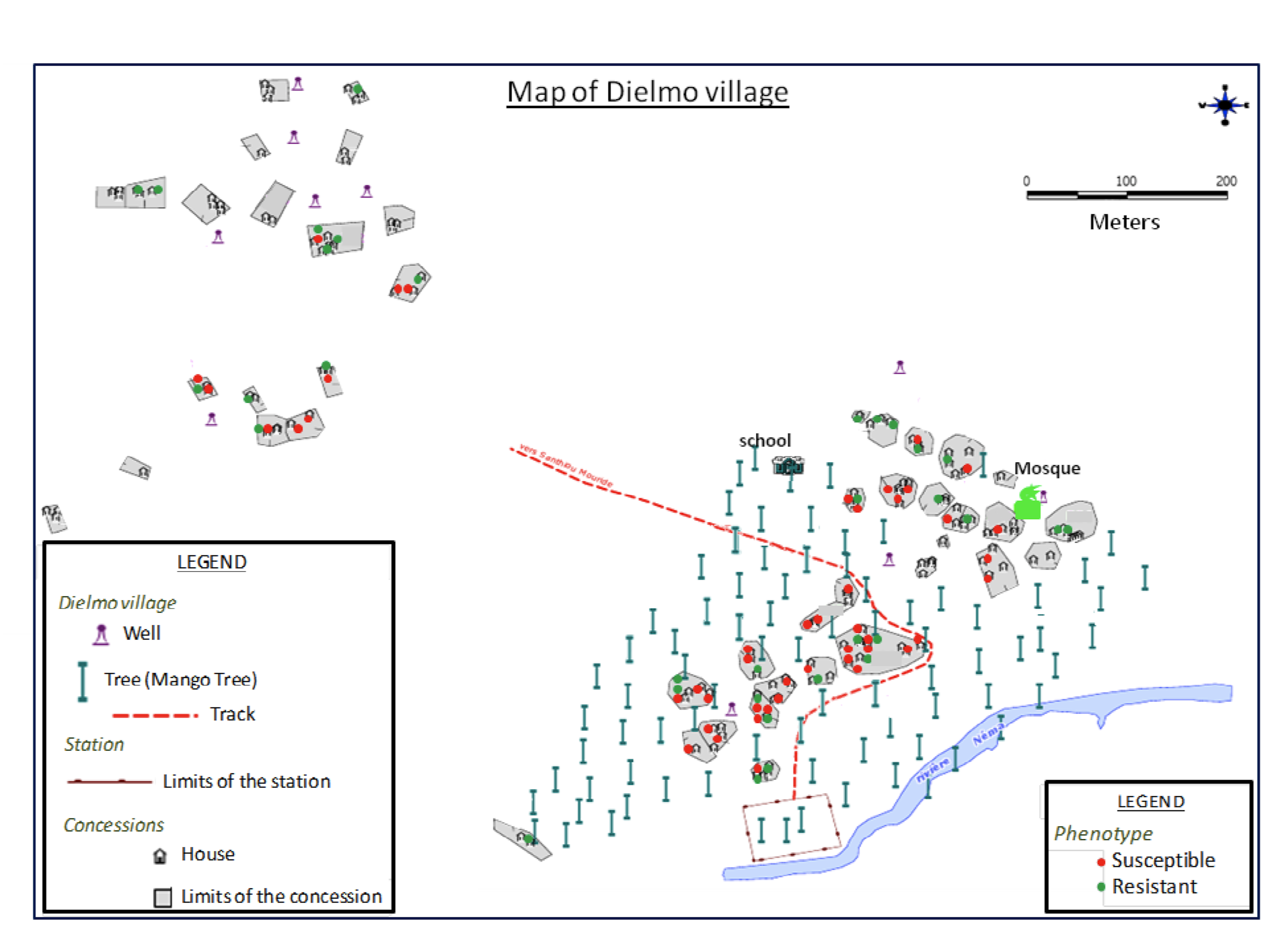

Supplement: Figure S7 — Localization of susceptible (red circle) and resistant (green circle) individuals as defined by “Age” inside Dielmo village. (TIF) [file pone.0055666.s007.tif]

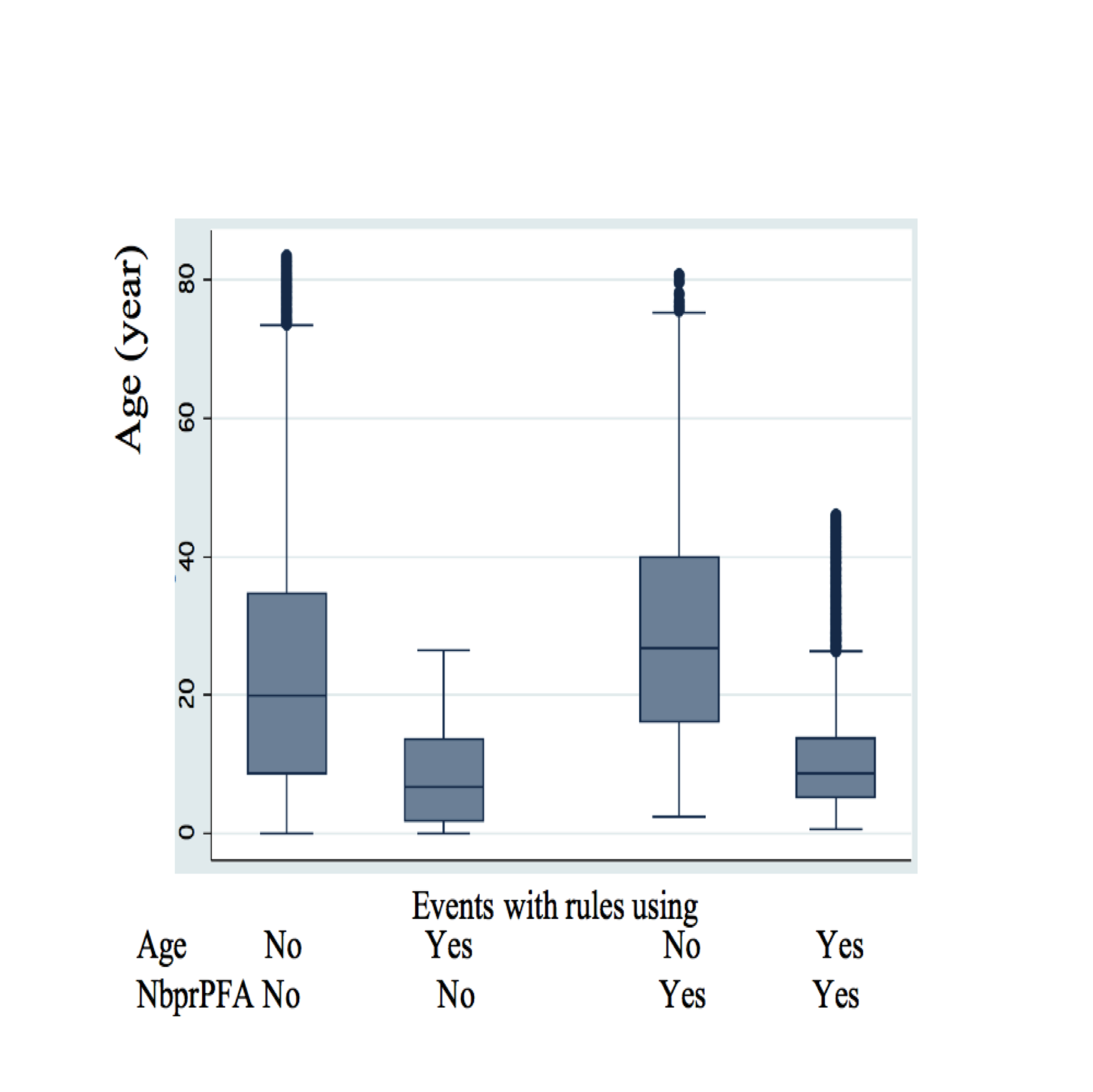

Supplement: Figure S8 — Age distribution of events defined by rules using either “Age” or “Number of previous PFA”, or both or neither in Ndiop village. After verifying by a one-way ANOVA that the four groups do not have identical mean ages (F(3,22510) = 1387.17; P<0.0001), the Scheffe test shows that each group has a mean age that is significantly different from the other (P<0.001). (TIF) [file pone.0055666.s008.tif]
