# Supplementary material for: High Number of Previous Plasmodium falciparum Clinical Episodes Increases Risk of Future Episodes in a Sub-Group of Individuals
Source: PLoS One. 2013 Feb 6;8(2):e55666. doi: 10.1371/journal.pone.0055666 (PMC3566008; doi:10.1371/journal.pone.0055666)
Supplement: Table S1 — Risk factors affecting clinical P. falciparum episodes in Dielmo village (All factors). (DOC) [file pone.0055666.s009.doc]

| Fixed effects | Estimate | Standard Error | z value | p-value |
| --- | --- | --- | --- | --- |
| Intercept | -1.70 | 0.40 | -4.21 | 2.58 10^-05^ |
| NbprPFA_1-2 | 0.91 | 0.14 | 6.61 | 3.94 10^-11^ |
| NbprPFA_3-5 | 1.55 | 0.14 | 10.81 | <2 10^-16^ |
| NbprPFA_6-9 | 2.07 | 0.15 | 13.92 | <2 10^-16^ |
| NbprPFA_10-12 | 2.17 | 0.17 | 12.62 | <2 10^-16^ |
| NbprPFA_13-16 | 2.29 | 0.18 | 13.06 | <2 10^-16^ |
| NbprPFA_17-21 | 2.52 | 0.18 | 13.92 | <2 10^-16^ |
| NbprPFA_22-27 | 2.57 | 0.18 | 13.66 | <2 10^-16^ |
| NbprPFA_28-34 | 2.22 | 0.21 | 10.85 | <2 10^-16^ |
| NbprPFA_35-45 | 2.62 | 0.22 | 11.82 | <2 10^-16^ |
| NbprPFA_46-55 | 2.31 | 0.25 | 9.12 | <2 10^-16^ |
| NbprPFA_56-89 | 2.77 | 0.30 | 9.15 | <2 10^-16^ |
| Age | -0.34 | 0.02 | -17.71 | <2 10^-16^ |
| Days of presence | 0.01 | 0.004 | 3.16 | 1.59 10^-03^ |

Note. Clinical *P. falciparum* episodes of all individuals born in the study were studied using the Generalized Linear Mixed Model with “NbprPFA_trim + Age + Days of presence” as fixed effects and “(1|individual) + (1|house) + (1|Drugperiod)” as random effects (Number of observation = 6513). Std. Dev._individual_ = 0.59 (n=285); Std. Dev._house_ = 0 (n=32); Std. Dev._Drugperiod_ = 0.20 (n=4). AIC = 6601; BIC = 6716; logLik = -3284. Figure S3 shows the distribution of residuals (Dielmo model 1).
