# Supplementary material for: High Number of Previous Plasmodium falciparum Clinical Episodes Increases Risk of Future Episodes in a Sub-Group of Individuals
Source: PLoS One. 2013 Feb 6;8(2):e55666. doi: 10.1371/journal.pone.0055666 (PMC3566008; doi:10.1371/journal.pone.0055666)
Supplement: Table S2 — Risk factors affecting clinical P. falciparum episodes in Dielmo village (Exclusion of Age). (DOC) [file pone.0055666.s010.doc]

| Fixed effects | Estimate | Standard Error | z value | p-value |
| --- | --- | --- | --- | --- |
| Intercept | -2.50 | 0.60 | -4.15 | 3.30 10^-05^ |
| NbprPFA_1-2 | 0.50 | 0.15 | 3.40 | 6.65 10^-04^ |
| NbprPFA_3-5 | 0.99 | 0.15 | 6.585 | 4.55 10^-11^ |
| NbprPFA_6-9 | 1.28 | 0.15 | 8.51 | < 2.0 10^-16^ |
| NbprPFA_10-12 | 0.97 | 0.16 | 5.915 | 3.32 10^-09^ |
| NbprPFA_13-16 | 0.96 | 0.17 | 5.79 | 7.12 10^-09^ |
| NbprPFA_17-21 | 0.91 | 0.17 | 5.50 | 3.80 10^-08^ |
| NbprPFA_22-27 | 0.78 | 0.17 | 4.61 | 3.97 10^-06^ |
| NbprPFA_28-34 | -0.02 | 0.18 | -0.12 | 0.90 |
| NbprPFA_35-45 | -0.11 | 0.18 | -0.59 | 0.56 |
| NbprPFA_46-55 | -0.99 | 0.21 | -4.78 | 1.76 10^-06^ |
| NbprPFA_56-89 | -1.27 | 0.26 | -4.95 | 7.61 10^-07^ |
| Days of presence | 0.01 | 0.004 | 3.59 | 3.33 10^--04^ |

Note. Clinical *P. falciparum* episodes of all individuals born in the study were studied using the Generalized Linear Mixed Model with “NbprPFA_trim + Days of presence” as fixed effects and “(1|individual) + (1|house) + (1|Drugperiod)” as random effects (Number of observation = 6513). Std. Dev._individual_ = 1.75 (n=285); Std. Dev._house_ = 1.08 10^-05^ (n=32); Std. Dev._Drugperiod_ = 0.95 (n=4). AIC = 6881; BIC = 6989; logLik = -3424. Figure S3 shows the distribution of residuals (Dielmo model 2).
