# Supplementary material for: High Number of Previous Plasmodium falciparum Clinical Episodes Increases Risk of Future Episodes in a Sub-Group of Individuals
Source: PLoS One. 2013 Feb 6;8(2):e55666. doi: 10.1371/journal.pone.0055666 (PMC3566008; doi:10.1371/journal.pone.0055666)
Supplement: Table S3 — Risk factors affecting clinical P. falciparum episodes in Dielmo village (Exclusion of NbprPFA). (DOC) [file pone.0055666.s011.doc]

| Fixed effects | Estimate | Standard Error | z value | p-value |
| --- | --- | --- | --- | --- |
| Intercept | -2.46 | 0.44 | -5.665 | 1.47 10^-08^ |
| Age | -0.18 | 0.01 | -14.51 | <2.0 10^-16^ |
| Days of presence | 0.02 | 0.004 | 6.91 | 4.82 10^-12^ |

Note. Clinical *P. falciparum* episodes of all individuals born in the study were studied using the Generalized Linear Mixed Model with “Age + Days of presence” as fixed effects and “(1|individual) + (1|house) + (1|Drugperiod)” as random effects (Number of observation = 6695). Std. Dev._individual_ = 1.28 (n=296); Std. _Dev.house_ = 3.96 10^-14^ (n=32); Std. Dev._Drugperiod_ = 0.30 (n=4). AIC = 7064; BIC = 7105; logLik = -3526. Figure S3 shows the distribution of residuals (Dielmo model 3).
