# Supplementary material for: High Number of Previous Plasmodium falciparum Clinical Episodes Increases Risk of Future Episodes in a Sub-Group of Individuals
Source: PLoS One. 2013 Feb 6;8(2):e55666. doi: 10.1371/journal.pone.0055666 (PMC3566008; doi:10.1371/journal.pone.0055666)
Supplement: Table S4 — Risk factors affecting clinical P. falciparum episodes in Ndiop village (All factors). (DOC) [file pone.0055666.s012.doc]

| Fixed effects | Estimate | Standard Error | z value | p-value |
| --- | --- | --- | --- | --- |
| Intercept | -3.68 | 0.48 | -7.74 | 1.0 10^-14^ |
| NbprPFA_1-2 | 0.85 | 0.15 | 5.81 | 6.20 10^-09^ |
| NbprPFA_3-5 | 1.36 | 0.15 | 9.03 | <2 10^-16^ |
| NbprPFA_6-9 | 1.73 | 0.17 | 10.36 | <2 10^-16^ |
| NbprPFA_10-12 | 2.32 | 0.20 | 11.41 | <2 10^-16^ |
| NbprPFA_13-16 | 2.25 | 0.21 | 10.58 | <2 10^-16^ |
| NbprPFA_17-21 | 2.10 | 0.22 | 9.32 | <2 10^-16^ |
| NbprPFA_22-27 | 2.36 | 0.26 | 9.18 | <2 10^-16^ |
| NbprPFA_28-59 | 2.53 | 0.29 | 8.85 | <2 10^-16^ |
| Age | -0.11 | 0.02 | -4.65 | 3.29 10^-06^ |
| Semester 2 | 3.08 | 0.09 | 33.56 | <2 10^-16^ |

Note. Clinical *P. falciparum* episodes of all individuals born in the study were studied using the Generalized Linear Mixed Model with “NbprPFA_trim + Age + Semester 2” as fixed effects and “(1|individual) + (1|house) + (1|Drugperiod)” as random effects (Number of observation = 5449). Std. Dev._indvidual_ = 0.13 (n=259); Std. Dev._house_ = 9.64 10^-03^ (n=26); Std. Dev._Drugperiod_ = 0.81 (n=4). AIC = 4626;BIC = 4718; logLik = -2299. Figure S3 shows the distribution of residuals (Ndiop model 1).
