# Supplementary material for: High Number of Previous Plasmodium falciparum Clinical Episodes Increases Risk of Future Episodes in a Sub-Group of Individuals
Source: PLoS One. 2013 Feb 6;8(2):e55666. doi: 10.1371/journal.pone.0055666 (PMC3566008; doi:10.1371/journal.pone.0055666)
Supplement: Table S5 — Risk factors affecting clinical P. falciparum episodes in Ndiop village (Exclusion of Age). (DOC) [file pone.0055666.s013.doc]

| Fixed effects | Estimate | Standard Error | z value | p-value |
| --- | --- | --- | --- | --- |
| Intercept | -3.80 | 0.54 | -7.10 | 1.25 10^-12^ |
| NbprPFA_1-2 | 0.69 | 0.14 | 4.84 | 1.28 10^-06^ |
| NbprPFA_3-5 | 1.12 | 0.14 | 7.87 | 3.42 10^-15^ |
| NbprPFA_6-9 | 1.34 | 0.14 | 9.30 | < 2.0 10^-16^ |
| NbprPFA_10-12 | 1.81 | 0.17 | 10.59 | < 2.0 10^-16^ |
| NbprPFA_13-16 | 1.64 | 0.17 | 9.75 | < 2.0 10^-16^ |
| NbprPFA_17-21 | 1.37 | 0.17 | 8.28 | < 2.0 10^-16^ |
| NbprPFA_22-27 | 1.54 | 0.19 | 7.95 | 1.87 10^-15^ |
| NbprPFA_28-59 | 1.45 | 0.19 | 7.85 | 4.10 10^-15^ |
| Semester 2 | 3.06 | 0.09 | 33.62 | < 2.0 10^-16^ |

Note. Clinical *P. falciparum* episodes of all individuals born in the study were studied using the Generalized Linear Mixed Model with “NbprPFA_trim + Semester 2” as fixed effects and “(1|individual) + (1|house) + (1|Drugperiod)” as a random effects (Number of observation = 5449). Std. Dev._individual_ = 0.18 (n=259); Std. Dev._house_ = 1.66 10^-02^ (n=26); Std. Dev._Drugperiod_ = 1.05 (n=4). AIC = 4644; BIC = 4730; logLik = -2309. Figure S3 shows the distribution of residuals (Ndiop model 2).
