# Supplementary material for: High Number of Previous Plasmodium falciparum Clinical Episodes Increases Risk of Future Episodes in a Sub-Group of Individuals
Source: PLoS One. 2013 Feb 6;8(2):e55666. doi: 10.1371/journal.pone.0055666 (PMC3566008; doi:10.1371/journal.pone.0055666)
Supplement: Table S6 — Risk factors affecting clinical P. falciparum episodes in Ndiop village (Exclusion of NbprPFA). (DOC) [file pone.0055666.s014.doc]

| Fixed effects | Estimate | Standard Error | z value | p-value |
| --- | --- | --- | --- | --- |
| Intercept | -3.42 | 0.52 | -6.59 | 4.38 10^-11^ |
| Age | 0.11 | 0.01 | 7.67 | 1.67 10^-14^ |
| Semester 2 | 2.89 | 0.09 | 33.38 | < 2.0 10^-16^ |

Note. Clinical *P. falciparum* episodes of all individuals born in the study were studied using the Generalized Linear Mixed Model with “Age + Semester 2” as fixed effects and “(1|individual) + (1|house) + (1|Drugperiod)” as random effects (Number of observation = 5708). Std. Dev._individual_ = 0.30 (n=264); Std. Dev._house_ = 3.11 10^-02^ (n=26); Std. Dev._Drugperiod_ = 1.00 (n=4). AIC = 4949; BIC = 4989; logLik = -2468. Figure S3 shows the distribution of residuals (Ndiop model 3).
