# Supplementary material for: High Number of Previous Plasmodium falciparum Clinical Episodes Increases Risk of Future Episodes in a Sub-Group of Individuals
Source: PLoS One. 2013 Feb 6;8(2):e55666. doi: 10.1371/journal.pone.0055666 (PMC3566008; doi:10.1371/journal.pone.0055666)
Supplement: Table S7 — Risk factors affecting clinical P. falciparum episodes in Dielmo village (All factors; Age analyzed as categories). (DOC) [file pone.0055666.s015.doc]

| Fixed effects | Estimate | Standard Error | z value | p-value |
| --- | --- | --- | --- | --- |
| Intercept | -2.02 | 0.43 | -4.66 | 3.14 10^-06^ |
| NbprPFA_1-2 | 0.61 | 0.14 | 4.51 | 6.47 10^-06^ |
| NbprPFA_3-5 | 1.13 | 0.14 | 7.90 | 2.89 10^-15^ |
| NbprPFA_6-9 | 1.56 | 0.15 | 10.14 | <2 10^-16^ |
| NbprPFA_10-12 | 1.58 | 0.18 | 8.87 | <2 10^-16^ |
| NbprPFA_13-16 | 1.69 | 0.19 | 9.11 | <2 10^-16^ |
| NbprPFA_17-21 | 1.88 | 0.19 | 9.74 | <2 10^-16^ |
| NbprPFA_22-27 | 1.94 | 0.20 | 9.67 | <2 10^-16^ |
| NbprPFA_28-34 | 1.59 | 0.21 | 7.39 | 1.44 10^-13^ |
| NbprPFA_35-45 | 1.91 | 0.22 | 8.61 | <2 10^-16^ |
| NbprPFA_46-55 | 1.60 | 0.25 | 6.41 | 1.49 10^-10^ |
| NbprPFA_56-89 | 1.90 | 0.29 | 6.58 | 4.58 10^-11^ |
| Age_3-5 | -0.53 | 0.12 | -4.58 | 4.68 10^-06^ |
| Age_6-8 | -1.77 | 0.15 | -11.71 | <2 10^-16^ |
| Age_9-11 | -2.59 | 0.18 | -14.17 | <2 10^-16^ |
| Age_12-14 | -3.00 | 0.24 | -12.73 | <2 10^-16^ |
| Age_15-19 | -4.35 | 0.51 | -8.52 | <2 10^-16^ |
| Days of presence | 0.01 | 0.004 | 3.18 | 1.43 10^-03^ |

Note Clinical *P. falciparum* episodes of all individuals born in the study were studied using the Generalized Linear Mixed Model with “NbprPFA_trim + Age + Days of presence” as fixed effects and “(1|individual) + (1|house) + (1|Drugperiod)” as random effects (Number of observation = 6513). Std. Dev._individual_ = 0.67 (n=285); Std. Dev._house_ = 0.0 (n=32); Std. Dev._Drugperiod_ = 0.29 (n=4). AIC = 6618; BIC = 4760; logLik = -3288.
