# Supplementary material for: High Number of Previous Plasmodium falciparum Clinical Episodes Increases Risk of Future Episodes in a Sub-Group of Individuals
Source: PLoS One. 2013 Feb 6;8(2):e55666. doi: 10.1371/journal.pone.0055666 (PMC3566008; doi:10.1371/journal.pone.0055666)
Supplement: Table S8 — Risk factors affecting clinical P. falciparum episodes in Dielmo village (Exclusion of NbprPFA; Age analyzed as categories). (DOC) [file pone.0055666.s016.doc]

| Fixed effects | Estimate | Standard Error | z value | p-value |
| --- | --- | --- | --- | --- |
| Intercept | -2.57 | 0.43 | -5.99 | 2.13 10^-09^ |
| Age_3-5 | 0.31 | 0.08 | 3.64 | 2.68 10^-04^ |
| Age_6-8 | -0.86 | 0.10 | -8.45 | < 2 10^-16^ |
| Age_9-11 | -1.64 | 0.13 | -12.47 | < 2 10^-16^ |
| Age_12-14 | -2.02 | 0.19 | -10.46 | < 2 10^-16^ |
| Age_15-19 | -3.33 | 0.49 | -6.83 | 8.41 10^-12^ |
| Days of presence | 0.02 | 0.004 | 5.90 | 3.60 10^-09^ |

Note. Clinical *P. falciparum* episodes of all individuals born in the study were studied using the Generalized Linear Mixed Model with “Age + Days of presence” as fixed effects and “(1|individual) + (1|house) + (1|Drugperiod)” as random effects (Number of observation = 6695). Std. Dev._individual_ = 1.12 (n=296); Std. _Dev.house_ = 1.71 10^-13^ (n=32); Std. Dev._Drugperiod_ = 0.30 (n=4). AIC = 6896; BIC = 6964; logLik = -3438.
