# Supplementary material for: High Number of Previous Plasmodium falciparum Clinical Episodes Increases Risk of Future Episodes in a Sub-Group of Individuals
Source: PLoS One. 2013 Feb 6;8(2):e55666. doi: 10.1371/journal.pone.0055666 (PMC3566008; doi:10.1371/journal.pone.0055666)
Supplement: Table S9 — Risk factors affecting clinical P. falciparum episodes in Ndiop village (All factors; Age analyzed as categories). (DOC) [file pone.0055666.s017.doc]

| Fixed effects | Estimate | Standard Error | z value | p-value |
| --- | --- | --- | --- | --- |
| Intercept | -3.79 | 0.49 | -7.76 | 8.74 10^-15^ |
| NbprPFA_1-2 | 0.70 | 0.14 | 4.89 | 1.02 10^-06^ |
| NbprPFA_3-5 | 1.12 | 0.15 | 7.44 | 1.04 10^-13^ |
| NbprPFA_6-9 | 1.38 | 0.17 | 7.94 | 2.00 10^-15^ |
| NbprPFA_10-12 | 1.95 | 0.21 | 9.31 | <2 10^-16^ |
| NbprPFA_13-16 | 1.90 | 0.21 | 8.90 | <2 10^-16^ |
| NbprPFA_17-21 | 1.80 | 0.22 | 8.17 | 3.08 10^-16^ |
| NbprPFA_22-27 | 2.11 | 0.25 | 8.43 | <2 10^-16^ |
| NbprPFA_28-59 | 2.32 | 0.26 | 8.82 | <2 10^-16^ |
| Age_3-5 | 0.08 | 0.13 | 0.61 | 0.53 |
| Age_6-8 | -0.49 | 0.17 | -2.82 | 4.82 10^-03^ |
| Age_9-11 | -0.75 | 0.23 | -3.34 | 8.53 10^-04^ |
| Age_12-16 | -1.50 | 0.32 | -4.75 | 2.07 10^-06^ |
| Semester 2 | 3.08 | 0.09 | 33.48 | <2 10^-16^ |

Note. Clinical *P. falciparum* episodes of all individuals born in the study were studied using the Generalized Linear Mixed Model with “NbprPFA_trim + Age + Semester 2” as fixed effects and “(1|individual) + (1|house) + (1|Drugperiod)” as random effects (Number of observation = 5449). Std. Dev._individual_ = 0.12 (n=259); Std. Dev._house_ = 1.57 10^-2^ (n=26); Std. Dev._Drugperiod_ = 0.86 (n=4). AIC = 4616; BIC = 4728; logLik = -2291.
