# Supplementary material for: High Number of Previous Plasmodium falciparum Clinical Episodes Increases Risk of Future Episodes in a Sub-Group of Individuals
Source: PLoS One. 2013 Feb 6;8(2):e55666. doi: 10.1371/journal.pone.0055666 (PMC3566008; doi:10.1371/journal.pone.0055666)
Supplement: Table S10 — Risk factors affecting clinical P. falciparum episodes in Ndiop village (Exclusion of NbprPFA; Age analyzed as categories). (DOC) [file pone.0055666.s018.doc]

| Fixed effects | Estimate | Standard Error | z value | p-value |
| --- | --- | --- | --- | --- |
| Intercept | -3.52 | 0.47 | -7.49 | 6.83 10^-14^ |
| Age_3-5 | 1.07 | 0.10 | 11.13 | <2 10^-16^ |
| Age_6-8 | 0.90 | 0.11 | 7.93 | 2.16 10^-15^ |
| Age_9-11 | 0.79 | 0.16 | 5.01 | 5.45 10^-07^ |
| Age_12-16 | 0.14 | 0.26 | 0.54 | 0.59 |
| Semester 2 | 2.96 | 0.09 | 33.37 | <2 10^-16^ |

Note. Clinical *P. falciparum* episodes of all individuals born in the study were studied using the Generalized Linear Mixed Model with “Age + Semester 2” as fixed effects and “(1|individual) + (1|house) + (1|Drugperiod)” as random effects (Number of observation = 5708). Std. Dev._individual_ = 0.32 (n=264); Std. Dev._house_ = 3.90 10^-02^ (n=26); Std. Dev._Drugperiod_ = 0.80 (n=4). AIC = 4866; BIC = 4926; logLik = -2424.
