# Supplementary material for: High Number of Previous Plasmodium falciparum Clinical Episodes Increases Risk of Future Episodes in a Sub-Group of Individuals
Source: PLoS One. 2013 Feb 6;8(2):e55666. doi: 10.1371/journal.pone.0055666 (PMC3566008; doi:10.1371/journal.pone.0055666)
Supplement: Table S11 — Risk factors affecting clinical P. falciparum episodes stratified according to Age in Dielmo village. (DOC) [file pone.0055666.s019.doc]

| **Age group** | **Variable** | **PFA (binary)** | **nbPFA (poisson)** |
| --- | --- | --- | --- |
| **(year)** |  | **Beta (p value)** | **Beta (p value)** |
| **00-02** | Intercept | -2.602 (3.8 10^-05^) | -2.3291 (1.7 10^-07^) |
|  | NbpreviousPFA_1 | 0.710 (2.6 10^-04^) | 0.5041 (1.8 10^-05^) |
|  | NbpreviousPFA _2 -3 | 0.910 (5.2 10^-07^) | 0.5954 (1.4 10^-08^) |
|  | NbpreviousPFA_4-14 | 1.581 (2.0 10^-15^) | 0.8137 (<2 10^-16^) |
|  | drugperiod 2 | 0.279 (1.3 10^-01^) | -0.0058 (9.5 10^-01^) |
|  | drugperiod 3 | -0.985 (4.9 10^-05^) | -0.7978 (1.5 10^-07^) |
|  | drugperiod 4 | -1.967 (3.2 10^-11^) | -1.8444 (8.2 10^-15^) |
|  | Days of presence | 0.022 (1.7 10^-03^) | 0.0185 (1.7 10^-04^) |
| **03-04** | Intercept | -0.2701 (7.5 10^-01^) | -0.6250 (1.2 10^-01^) |
|  | NbpreviousPFA_4-8 | 0.7345 (6.4 10^-04^) | 0.3766 (9.4 10^-04^) |
|  | NbpreviousPFA_9-14 | 1.2016 (1.8 10^-06^) | 0.5802 (8.1 10^-07^) |
|  | NbpreviousPFA_15-37 | 1.6549 (1.7 10^-08^) | 0.7497 (6.3 10^-10^) |
|  | drugperiod 2 | 0.2047 (4.7 10^-01^) | -0.0220 (8.3 10^-01^) |
|  | drugperiod 3 | -0.8771 (6.9 10^-03^) | -0.6125 (2.9 10^-06^) |
|  | drugperiod 4 | -1.5045 (6.8 10^-06^) | -1.1035 (2.1 10^-12^) |
|  | Days ofpresence | 0.0033 (7.2 10^-01^) | 0.0045 (3.0 10^-01^) |
| **05-07** | Intercept | -0.727 (4.1 10^-01^) | -0.767 (1.1 10^-01^) |
|  | NbpreviousPFA_11-19 | 0.530 (9.9 10^-03^) | 0.376 (2.2 10^-03^) |
|  | NbpreviousPFA_20-28 | 0.744 (8.1 10^-04^) | 0.445 (5.2 10^-04^) |
|  | NbpreviousPFA_ 29-58 | 0.927 (1.1 10^-04^) | 0.535 (6.2 10^-05^) |
|  | drugperiod 2 | -0.650 (2.9 10^-01^) | -0.579 (1.8 10^-02^) |
|  | drugperiod 3 | -1.650 (9.4 10^-03^) | -1.337 (3.1 10^-07^) |
|  | drugperiod 4 | -1.955 (2.1 10^-03^) | -1.524 (1.1 10^-08^) |
|  | Days of presence | 0.015 (4.2 10^-02^) | 0.011 (2.2 10^-02^) |
| **08-10** | Intercept | -0.8874 (2.9 10^-01^) | -0.9456 (7.5 10^-02^) |
|  | NbpreviousPFA_16-25 | 0.1358 (7.2 10^-01^) | -0.0069 (9.8 10^-01^) |
|  | NbpreviousPFA_26-40 | 0.7793 (4.3 10^-02^) | 0.5922 (3.7 10^-02^) |
|  | NbpreviousPFA_41-73 | 1.2200 (2.8 10^-03^) | 0.8780 (3.0 10^-03^) |
|  | drugperiod 3 | -0.5923 (1.6 10^-02^) | -0.5117 (3.1 10^-03^) |
|  | drugperiod 4 | -1.2503 (1.4 10^-05^) | -1.1194 (3.1 10^-07^) |
|  | Days of presence | -0.0075 (4.1 10^-01^) | -0.0074 (2.0 10^-01^) |
| **11-19** | Intercept | -5.919 (9.9 10^-06^) | -5.491 (5.5 10^-06^) |
|  | NbpreviousPFA_17-34 | 0.750 (1.4 10^-01^) | 0.613 (1.6 10^-01^) |
|  | NbpreviousPFA_35-50 | 0.714 (2.2 10^-01^) | 0.654 (1.9 10^-01^) |
|  | NbpreviousPFA_51-89 | 1.363 (2.6 10^-02^) | 1.286 (1.3 10^-02^) |
|  | drugperiod 3 | -1.069 (3.6 10^-05^) | -0.937 (3.1 10^-06^) |
|  | drugperiod 4 | -1.490 (3.6 10^-07^) | -1.258 (9.4 10^-08^) |
|  | Days of presence | 0.039 (5.0 10^-03^) | 0.033 (8.0 10^-03^) |

Note. Clinical *P. falciparum* episodes of all individuals born in the study were studied using the Generalized Linear Mixed Model with “NbpreviousPFA + Drug period + Days of presence” as fixed effects and “(1|individual) + (1|house)” as random effects.
