# Supplementary material for: High Number of Previous Plasmodium falciparum Clinical Episodes Increases Risk of Future Episodes in a Sub-Group of Individuals
Source: PLoS One. 2013 Feb 6;8(2):e55666. doi: 10.1371/journal.pone.0055666 (PMC3566008; doi:10.1371/journal.pone.0055666)
Supplement: Table S12 — Risk factors affecting clinical P. falciparum episodes stratified according to Age in Ndiop village. (DOC) [file pone.0055666.s020.doc]

| **Age group** | **Variable** | **PFA (binary)** | **nbPFA (poisson)** |
| --- | --- | --- | --- |
| **(year)** |  | **Beta_(p value)** | **Beta_(p value)** |
| **00-01** | Intercept | -3.17 (1.8 10^-11^) | -3.07 (2.2 10^-16^) |
|  | NbpreviousPFA_1-5 | 1.38 (1.5 10^-05^) | 0.89 (2.1 10^-07^) |
|  | drugperiod 2 | -0.23 (5.5 10^-01^) | -0.12 (6.5 10^-01^) |
|  | drugperiod 3 | -0.72 (1.2 10^-01^) | -0.36 (2.6 10^-01^) |
|  | drugperiod 4 | -2.45 (3.9 10^-05^) | -2.14 (3.2 10^-05^) |
|  | semester 2 | 2.91 (2.2 10^-16^) | 2.41 (2.2 10^-16^) |
| **02-03** | Intercept | -2.22 (1.3 10^-06^) | -2.41 (<2 10^-16^) |
|  | NbpreviousPFA_1-2 | 0.45 (4.6 10^-02^) | 0.41 (1.9 10^-03^) |
|  | NbpreviousPFA_3_4 | 0.60 (1.6 10^-02^) | 0.43 (2.8 10-^03^) |
|  | NbpreviousPFA_5-15 | 0.92 (7.0 10^-04^) | 0.67 (4.1 10^-06^) |
|  | drugperiod 2 | -0.72 (8.5 10^-02^) | -0.22 (2.7 10^-01^) |
|  | drugperiod 3 | -0.91 (4.4 10^-02^) | -0.21 (3.3 10^-01^) |
|  | drugperiod 4 | -2.95 (8.3 10^-10^) | -1.83 (1.6 10^-11^) |
|  | semester 2 | 3.06 (<2 10^-16^) | 2.22 (<2 10-16) |
| **04-05** | Intercept | -2.61 (<2 10^-16^) | -1.90 (<2 10^-16^) |
|  | NbpreviousPFA_4-7 | 0.63 (7.6 10^-03^) | 0.33 (8.4 10^-03^) |
|  | NbpreviousPFA_8-10 | 1.02 (1.0 10^-04^) | 0.39 (3.4 10^-03^) |
|  | NbpreviousPFA_11-22 | 1.69 (1.3 10^-09^) | 0.68 (2.3 10^-07^) |
|  | drugperiod 3 | -0.59 (4.8 10^-03^) | -0.21 (2.7 10^-02^) |
|  | drugperiod 4 | -2.41 (<2 10^-16^) | -1.76 (<2 10^-16^) |
|  | semester 2 | 3.26 (<2 10^-16^) | 1.88 (<2 10^-16^) |
| **04-08** | Intercept | -2.17 (<2 10^-16^) | -1.94 (<2 10^-16^) |
|  | NbpreviousPFA_10-14 | 0.65 (2.9 10^-03^) | 0.43 (2.2 10^-04^) |
|  | NbpreviousPFA_15-19 | 0.73 (1.1 10^-03^) | 0.48 (4.4 10^-05^) |
|  | NbpreviousPFA_20-35 | 1.03 (1.2 10^-05^) | 0.53 (1.3 10^-05^) |
|  | drugperiod 3 | -1.01 (5.8 10^-08^) | -0.46 (1.9 10^-07^) |
|  | drugperiod 4 | -2.24 (<2 10^-16^) | -1.40 (<2 10^-16^) |
|  | semester 2 | 3.07 (<2 10^-16^) | 1.97 (<2 10^-16^) |
| **09-16** | Intercept | -2.285 (1.0 10^-10^) | -2.36 (<2 10^-16^) |
|  | NbpreviousPFA_17-23 | -0.068 (8.0 10^-01^) | 0.01 (9.5 10^-01^) |
|  | NbpreviousPFA_24-32 | 0.713 (1.3 10^-02^) | 0.49 (4.9 10^-03^) |
|  | NbpreviousPFA_33-59 | 0.730 (1.5 10^-02^) | 0.42 (1.9 10^-02^) |
|  | drugperiod 3 | -1.058 (5.8 10^-04^) | -0.64 (3.0 10^-06^) |
|  | drugperiod 4 | -2.554 (4.4 10^-16^) | -1.66 (<2 10^-16^) |
|  | semester 2 | 3.388 (<2 10^-16^) | 2.51 (<2 10^-16^) |

Note. Clinical *P. falciparum* episodes of all individuals born in the study were studied using the Generalized Linear Mixed Model with “NbpreviousPFA + Drug period + Semester 2” as fixed effects and “(1|individual) + (1|house)” as a random effects.
