# Supplementary material for: High Number of Previous Plasmodium falciparum Clinical Episodes Increases Risk of Future Episodes in a Sub-Group of Individuals
Source: PLoS One. 2013 Feb 6;8(2):e55666. doi: 10.1371/journal.pone.0055666 (PMC3566008; doi:10.1371/journal.pone.0055666)
Supplement: Table S13 — Factors affecting maximal asymptomatic parasite density during a trimester. (DOC) [file pone.0055666.s021.doc]

| Factor** | Coefficient | Standard Error | Probability |
| --- | --- | --- | --- |
| Age | -3.077 | 0.693 | < 0.001 |
| NbprPFA | 0.485 | 0,169 | 0.004 |
| Drug treat. period 2 | 18.895 | 7.972 | 0.018 |
| Drug treat. period 3 | -13.257 | 8.801 | 0.132 |
| Drug treat. period 4 | -25.986 | 8.885 | 0.003 |
| Constant | 56.08 | 7.32 | < 0.001 |

Note. The maximum asymptomatic parasite density during a trimester was chosen as the dependent variable. Asymptomatic events were defined as those with no PFA during a trimester. A multilevel mixed-effects linear regression was performed using xtmixed function of Stata/IC 10.1 with three fixed effects, age (continuous), drug treatment period (ordered), and number of previous PFA (continuous) and one random effect, the individual effect with all variances-covariances directly estimated. There were 2,745 trimester-events for 282 individuals living in Dielmo village and born after the beginning of the project.

* A mixed-effect linear regression on people living in Dielmo village and born during the project.

** individual as random effect with variances-covariances distinctly estimated
