# Supplementary material for: High Number of Previous Plasmodium falciparum Clinical Episodes Increases Risk of Future Episodes in a Sub-Group of Individuals
Source: PLoS One. 2013 Feb 6;8(2):e55666. doi: 10.1371/journal.pone.0055666 (PMC3566008; doi:10.1371/journal.pone.0055666)
Supplement: Summary S1 — (DOC) [file pone.0055666.s022.doc]

**Supporting Information Legends**

*Summary of the first 12^th^ supplementary tables*

Table S1 to S3 tested the confounding effect of “Age” as a continuous variable in Dielmo village. Table S1 used all the variables; Table S2 gave results of a similar analysis after excluding “Age”, and Table S3 after excluding “NbprPFA”. Table S4 to S6 showed similar results to those of Table S1 to S3 for Ndiop village. Note also that taking “Drug period” variable as either a fixed (Table 5) or a random (Table S1) effect did not modify the results in particular that of “NbprPFA” variable. Data of Tables S4 and Table 6 confirmed this result for Ndiop village.

Table S7 using all the variables and Table S8 after excluding “NbprPFA” tested the confounding effect of “Age” as a categorical variable in Dielmo village. Note that except for the “Age” variable, all other variables are identical respectively to those of Table S1 and S3. Table S9 and S10 showed similar results to those of Table S7 and S8 for Ndiop village.

Table S11 gave results of analyses stratified according to “Age” variable in Dielmo village. Table S12 gave similar results for Ndiop village. In these last analyses, the dependent variable PFA was coded either as a binary trait (as previously) or as a Poisson trait.

Table S1: **Risk factors affecting clinical *P. falciparum* episodes in Dielmo village (All factors)**

Table S2: **Risk factors affecting clinical *P. falciparum* episodes in Dielmo village (Exclusion of Age)**

Table S3: **Risk factors affecting clinical *P. falciparum* episodes in Dielmo village (Exclusion of NbprPFA)**

Table S4: **Risk factors affecting clinical *P. falciparum* episodes in Ndiop village (All factors)**

Table S5: **Risk factors affecting clinical *P. falciparum* episodes in Ndiop village (Exclusion of Age)**

Table S6: **Risk factors affecting clinical *P. falciparum* episodes in Ndiop village (Exclusion of NbprPFA)**

Table S7: **Risk factors affecting clinical *P. falciparum* episodes in Dielmo village (All factors; Age analyzed as categories)**

Table S8: **Risk factors affecting clinical *P. falciparum* episodes in Dielmo village (Exclusion of NbprPFA; Age analyzed as categories)**

Table S9: **Risk factors affecting clinical *P. falciparum* episodes in Ndiop village (All factors; Age analyzed as categories)**

Table S10: **Risk factors affecting clinical *P. falciparum* episodes in Ndiop village (Exclusion of NbprPFA; Age analyzed as categories)**

Table S11: **Risk factors affecting clinical *P. falciparum* episodes stratified according to Age in Dielmo village**

Table S12: **Risk factors affecting clinical *P. falciparum* episodes stratified according to Age in Ndiop village**

Table S13: **Factors affecting maximal asymptomatic parasite density during a trimester**

Figure S1 **Distribution of residuals from model described in Table 5.**

Figure S2 **Distribution of residuals from model described in Table 6.**

Figure S3 **Distribution of residuals from models described in Table S1 to S6.**

Figure S4: **Histogram of Number of PFA during a trimester for individuals born during the project and living in Dielmo village before ACT therapy.** A: Age < 3 and NbprPFA < 5; B: Age < 3 and NbprPFA >= 5; C: 3<= Age < 6 and NbprPFA <20; D: 3<= Age < 6 and NbprPFA >= 20; E: 6 <= Age < 9 and NbprPFA < 35; F: 6 <= Age < 9 and NbprPFA >= 35; G: Age >= 9 and NbprPFA < 50; H: Age >= 9 and NbprPFA >= 50.

Figure S5: **Histogram of Number of PFA during a trimester for individuals born during the project and living in Ndiop village during the rainy season before ACT therapy.** A: Age < 3 and NbprPFA < 3; B: Age < 3 and NbprPFA >= 3; C: 3<= Age < 6 and NbprPFA <10; D: 3<= Age < 6 and NbprPFA >= 10; E: 6 <= Age < 9 and NbprPFA < 20; F: 6 <= Age < 9 and NbprPFA >= 20; G: Age >= 9 and NbprPFA < 45; H: Age >= 9 and NbprPFA >= 45.

Figure S6 **Localization of susceptible (red circle) and resistant (green circle) individuals as defined by “Number of previous PFA” inside Dielmo village.**

Figure S7 **Localization of susceptible (red circle) and resistant (green circle) individuals as defined by “Age” inside Dielmo village.**

Figure S8 **Age distribution of events defined by rules using either “Age” or “Number of previous PFA”, or both or neither in Ndiop village.** After verifying by a one-way ANOVA that the four groups do not have identical mean ages (F(3,22510) = 1387.17; *P* < 0.0001), the Scheffe test shows that each group has a mean age that is significantly different from the other (*P* < 0.001).
